# Supplementary material for: Quantitative Temporal Proteomic Analysis of Vaccinia Virus Infection Reveals Regulation of Histone Deacetylases by an Interferon Antagonist
Source: Cell Rep. 2019 May 7;27(6):1920–1933.e7. doi: 10.1016/j.celrep.2019.04.042 (PMC6518873; doi:10.1016/j.celrep.2019.04.042)
Supplement: Document S1. Figures S1–S5 [file mmc1.pdf]

Cell Reports, Volume *27*

Supplemental Information

Quantitative Temporal Proteomic Analysis of Vaccinia Virus Infection Reveals Regulation of Histone Deacetylases by an Interferon Antagonist

Lior Soday, Yongxu Lu, Jonas D. Albarnaz, Colin T.R. Davies, Robin Antrobus, Geoffrey L. Smith, and Michael P. Weekes

Figure S1

A

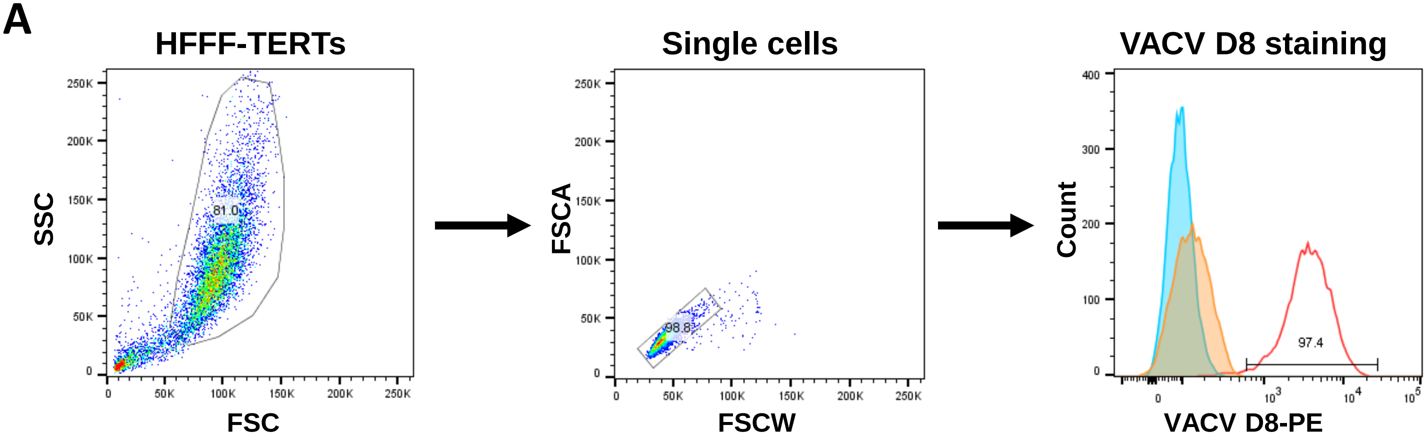

B

	WCL1	WCL2	WCL3	Any WCL	All WCL	MG / C6 data
**Total peptides**	120345	124779	112310			123980
**Human proteins**	8229	8310	8076	8991	7316	8263
**Viral proteins**	166	169	169	172	160	173

C

	**Sensitive Criteria** (average fold change of 2 at any time point)	**Stringent Criteria** (quantified in WCL1, 2 and 3; average fold change of 2 and a p <0.05 at any time point)
**Total Proteins**	8991	7316
**Downregulated**	265	142
**Upregulated**	70	4

D

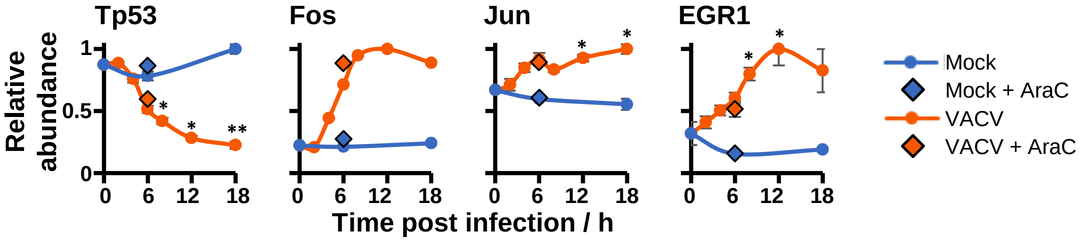

E

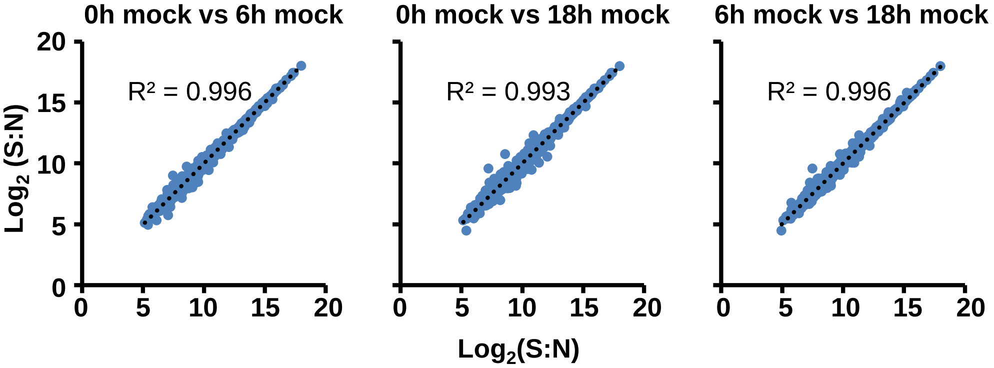

Figure S1. Technical aspects of the expreiments. related to Figures 1-5.

(A) Flow cytometry of HFFF-TERTs 12 h post infection using a mAb against late protein D8 (Parkinson and Smith, 1994) confirmed > 95% of cells were infected. A total 10,000 events were recorded, followed by sequential gating of HFFF-TERTs and single-cell populations. Isotype antibody staining of infected cells and D8 staining of non-infected cells were used to define the boundaries between "D8-positive" and "D8-negative" cells. Data shown are from experiment 1, and are representative of all three replicates.

(B) Peptides and proteins quantified in each experiment in this manuscript.

(C) Number of proteins that changed by sensitive or stringent criteria (see Figure 1C for definition of these criteria).

(D) Validation of down- or up-regulation of proteins already reported to be modulated by VACV. Data are represented as mean ± SEM; *p<0.05, *p<0.01 (see STAR Methods). Error bars and statistics are not included on the plot for Fos as this protein was not quantified in all three replicates; full data is shown in Table S1.

(E) Correspondence between mock samples. Each graph shows $\log_2$(normalised signal:noise) for every protein assessed in all three biological replicates.

Figure S2

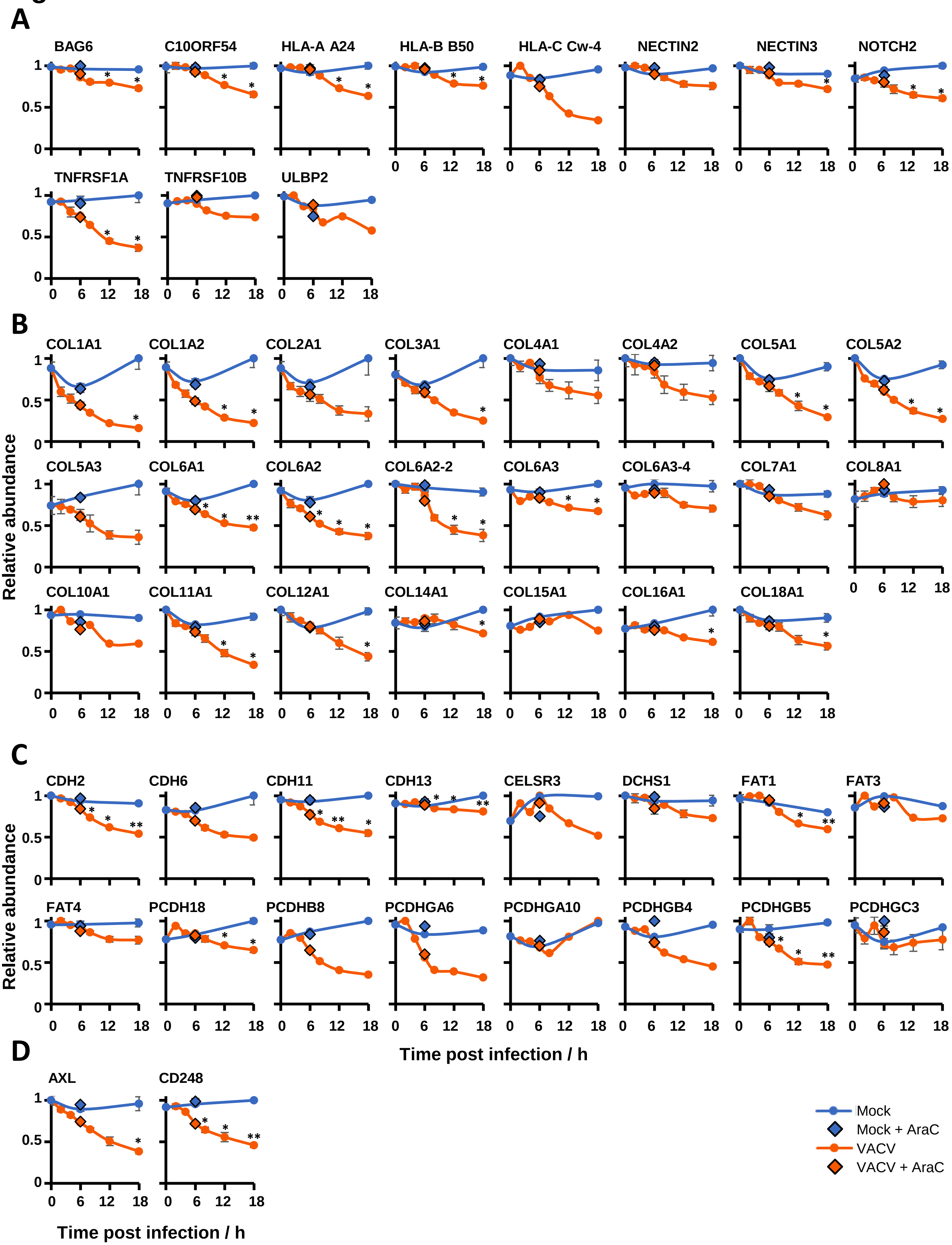

A
BAG6
C10ORF54
HLA-A A24
HLA-B B50
HLA-C Cw-4
NECTIN2
NECTIN3
NOTCH2
TNFRSF1A
TNFRSF10B
ULBP2
B
COL1A1
COL1A2
COL2A1
COL3A1
COL4A1
COL4A2
COL5A1
COL5A2
COL5A3
COL6A1
COL6A2
COL6A2-2
COL6A3
COL6A3-4
COL7A1
COL8A1
COL10A1
COL11A1
COL12A1
COL14A1
COL15A1
COL16A1
COL18A1
C
CDH2
CDH6
CDH11
CDH13
CELSR3
DCHS1
FAT1
FAT3
FAT4
PCDH18
PCDHB8
PCDHGA6
PCDHGA10
PCDHGB4
PCDHGB5
PCDHGC3
D
AXL
CD248
Relative abundance
Time post infection / h
Mock
Mock + AraC
VACV
VACV + AraC

Figure S2, Modulation of known and putative immune ligands by VACV infection, related to Figure 2. Data are represented as mean ± SEM; *p<0.05, *p<0.01 (see STAR Methods). In this figure, error bars and statistics are not included on the plots for HLA-C, TNFRSF10B, ULBP2, COL10A1, COL15A1, CELSR3, FAT3, PCDHB8, PCDHGA6, PCDHGA10, PCDHGB4 as these proteins were not quantified in all three replicates; full data is shown in Table S1.

(A) Modulation of known NK- or T-cell ligands.
(B) All collagens quantified.
(C) All protocadherins quantified.
(D) Downregulation of AXL and CD248.

Figure S3

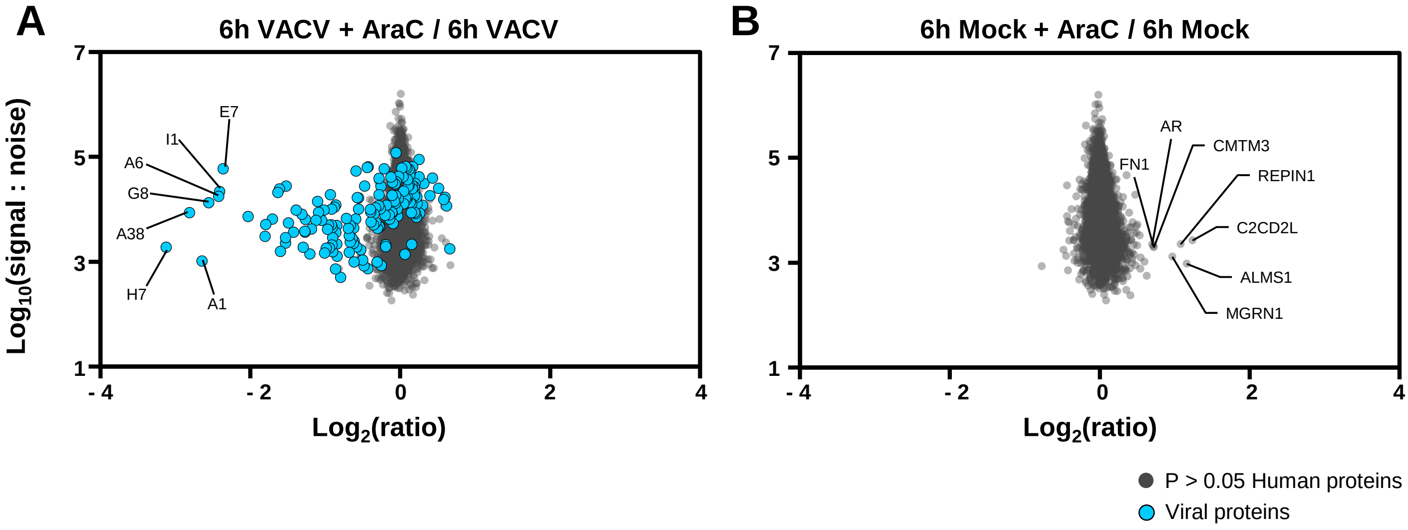

Figure S3. AraC inhibits expression of a subset of viral proteins, related to Figure 4.

(A) Scatter plot of all proteins quantified at 6 h of infection in all three replicates in the presence or absence of AraC. A Benjamini-Hochberg-corrected two-tailed t-test was used to estimate p-values.

(B) Scatter plot of all proteins quantified at 6 h of mock infection in the presence or absence of AraC. VACV proteins were quantified at the level of noise in mock samples, and are not shown.

Figure S4

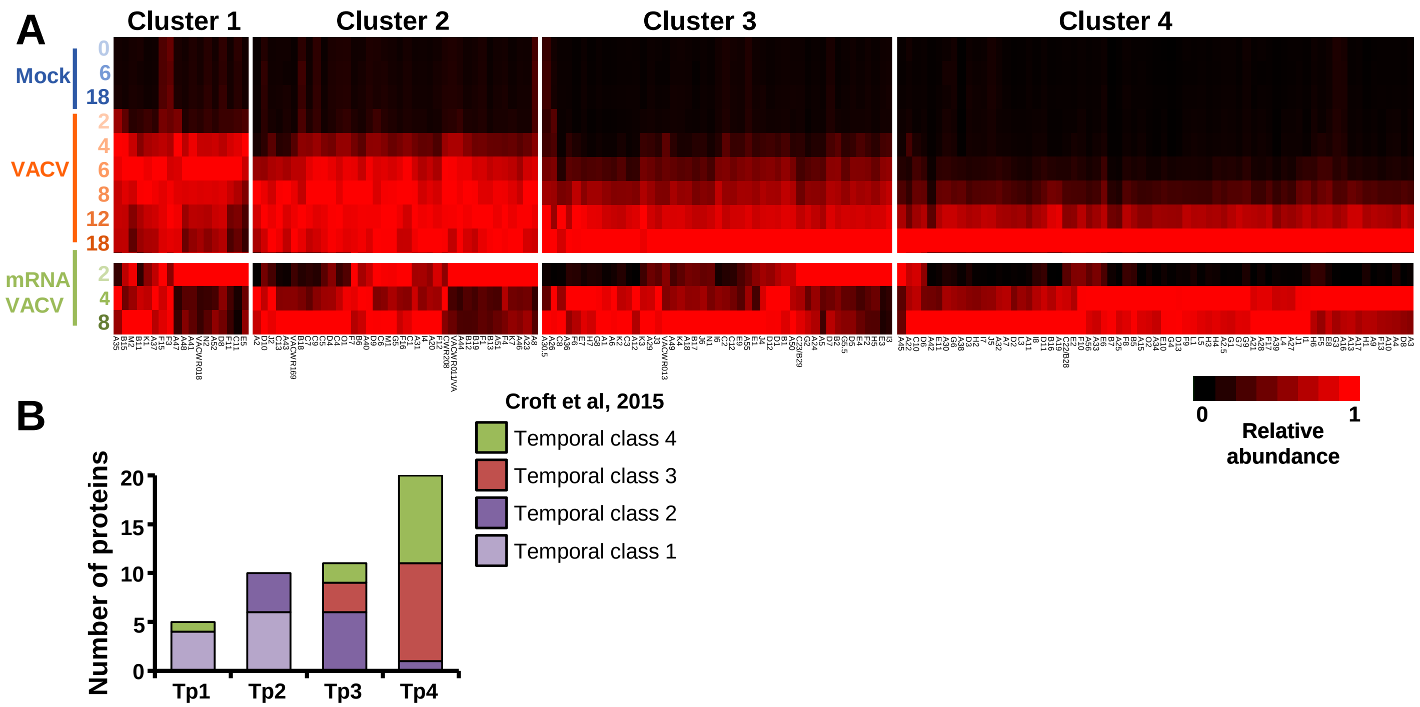

Figure S4. Comparison of viral protein data to previous studies, related to Figure 4.

(A) Comparison between temporal protein profiles (this study) and transcript expression profiles (Yang et al., 2015), grouped according to protein class. For each protein or transcript, expression was normalised to the maximum across the measured time points.

(B) Comparison of viral protein classes between this study and (Croft et al., 2015). Full data and an explanation of the consensus between the two time courses conducted by Croft et al., is included in Table S5.

Figure S5

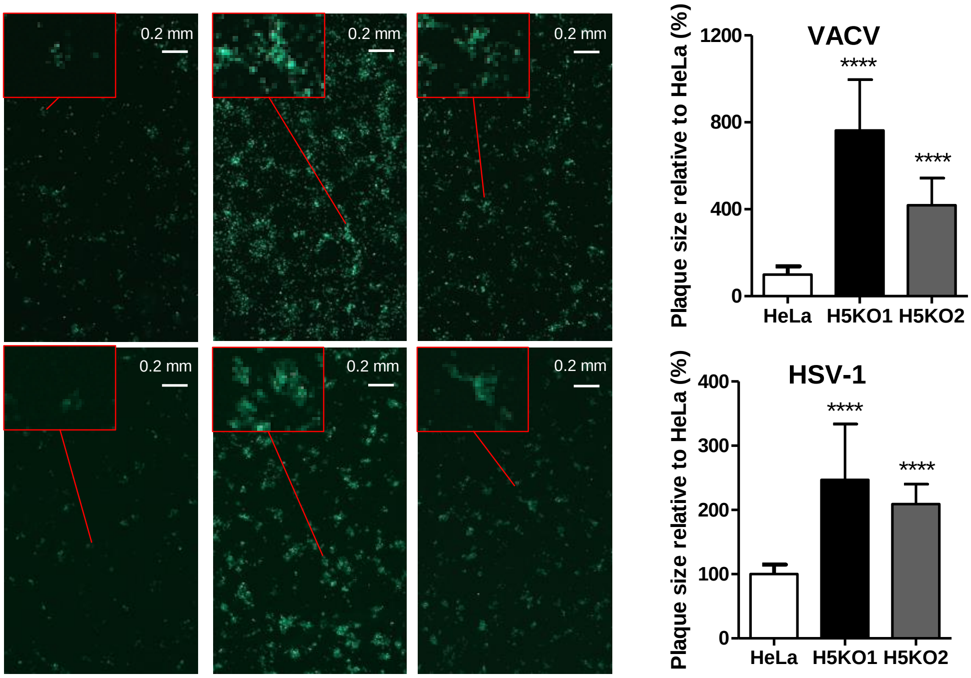

Figure S5. Enhanced spread of VACV and HSV-1 in HeLa H5KO1 and H5KO2 cells, related to Figure 7. Cells were infected with A5GFP VACV (for 2 d and VP26GFP HSV-1 for 3 d (MOI=0.001) and then imaged by fluorescence microscopy. The size of GFP infected foci (n=20) were measured for each virus and quantified as described in Materials and Methods. p-values were calculated using a two-tailed t-test. ****p<0.0001. The images shown are representative of 5 independent experiments.
