# Supplementary material for: Quantitative Temporal Proteomic Analysis of Vaccinia Virus Infection Reveals Regulation of Histone Deacetylases by an Interferon Antagonist
Source: Cell Rep. 2019 May 7;27(6):1920–1933.e7. doi: 10.1016/j.celrep.2019.04.042 (PMC6518873; doi:10.1016/j.celrep.2019.04.042)
Supplement: Document S2. Article plus Supplemental Information [file mmc9.pdf]

Resource

Cell Reports

Quantitative Temporal Proteomic Analysis of Vaccinia Virus Infection Reveals Regulation of Histone Deacetylases by an Interferon Antagonist

Graphical Abstract

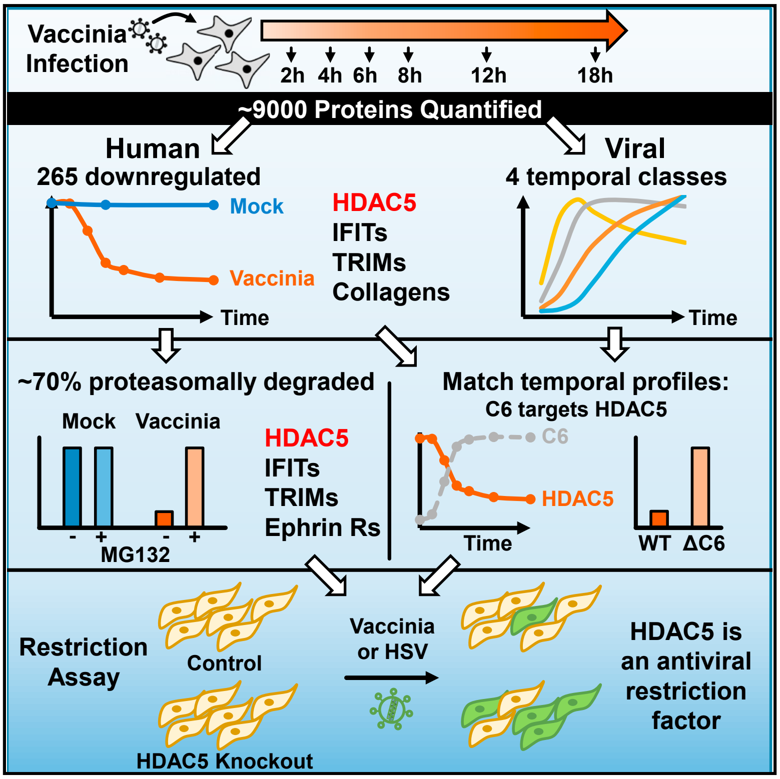

Highlights

- Temporal proteomic analysis quantifies host and viral dynamics during vaccinia infection
- Host protein families are proteasomally degraded over the course of vaccinia infection
- Vaccinia protein C6 targets HDAC5 for proteasomal degradation
- HDAC5 is a host antiviral factor that restricts different families of DNA viruses

Authors

Lior Soday, Yongxu Lu, Jonas D. Albarnaz, Colin T.R. Davies, Robin Antrobus, Geoffrey L. Smith, Michael P. Weekes

Correspondence

gls37@cam.ac.uk (G.L.S.), mpw1001@cam.ac.uk (M.P.W.)

In Brief

Soday et al. describe a temporal proteomic analysis of vaccinia virus infection that includes systematic investigation into virally induced host protein degradation. Vaccinia degrades multiple families of immune ligands and interferon-stimulated genes. The viral C6 protein targets histone deacetylase 5 (HDAC5) for proteasomal degradation to evade the antiviral activity of HDAC5.

Soday et al., 2019, Cell Reports *27*, 1920–1933
May 7, 2019 © 2019 The Author(s).
https://doi.org/10.1016/j.celrep.2019.04.042

CellPress

OPEN ACCESS
CellPress

Cell Reports
Resource

Quantitative Temporal Proteomic Analysis of Vaccinia Virus Infection Reveals Regulation of Histone Deacetylases by an Interferon Antagonist

Lior Soday,[1,3] Yongxu Lu,[2,3] Jonas D. Albarnaz,[2,3] Colin T.R. Davies,[1] Robin Antrobus,[1] Geoffrey L. Smith,[2,4,*] and Michael P. Weekes[1,4,5,*]
[1]Cambridge Institute for Medical Research, University of Cambridge, Hills Road, Cambridge CB2 0XY, UK
[2]Department of Pathology, University of Cambridge, Tennis Court Road, Cambridge CB2 1QP, UK
[3]These authors contributed equally
[4]Senior author
[5]Lead Contact
*Correspondence: gls37@cam.ac.uk (G.L.S.), mpw1001@cam.ac.uk (M.P.W.)
https://doi.org/10.1016/j.celrep.2019.04.042

SUMMARY

Vaccinia virus (VACV) has numerous immune evasion strategies, including multiple mechanisms of inhibition of interferon regulatory factor 3 (IRF-3), nuclear factor κB (NF-κB), and type I interferon (IFN) signaling. Here, we use highly multiplexed proteomics to quantify ~9,000 cellular proteins and ~80% of viral proteins at seven time points throughout VACV infection. A total of 265 cellular proteins are downregulated >2-fold by VACV, including putative natural killer cell ligands and IFN-stimulated genes. Two-thirds of these viral targets, including class II histone deacetylase 5 (HDAC5), are degraded proteolytically during infection. In follow-up analysis, we demonstrate that HDAC5 restricts replication of both VACV and herpes simplex virus type 1. By generating a protein-based temporal classification of VACV gene expression, we identify protein C6, a multifunctional IFN antagonist, as being necessary and sufficient for proteasomal degradation of HDAC5. Our approach thus identifies both a host antiviral factor and a viral mechanism of innate immune evasion.

INTRODUCTION

Vaccinia virus (VACV) is a large double-stranded DNA orthopoxvirus genetically related to variola virus, the causative agent of smallpox (Moss, 2013). The antigenic relatedness between orthopoxviruses leads to cross-protection to heterologous infections and explains why VACV was an effective live vaccine against smallpox, which culminated in the eradication of this disease in 1980 (Fenner et al., 1988). Following the eradication of smallpox, research with VACV has continued because it is an excellent model for studying virus-host interactions, and because VACV is being developed as a vector for live vaccines against other infectious diseases and as an oncolytic agent. To improve the safety of VACV as a vaccine and to optimize its immunogenicity, there is a need to have a comprehensive understanding of the biology of VACV and its interactions with the host cell.

VACV gene expression is conventionally divided into early, intermediate, and late phases (Moss, 2013). Early genes encode multiple proteins that suppress innate and adaptive host immunity, in addition to factors that initiate DNA replication and regulate intermediate class gene expression (Hruby and Ball, 1982; Jones and Moss, 1984; Sanz and Moss, 1999; Smith et al., 1989a, 1989b; Smith et al., 2013). Intermediate genes chiefly encode transcription factors that regulate late gene expression. Late genes encode structural proteins, as well as other proteins incorporated into progeny virions including viral RNA transcription machinery and early gene transcription factors (Broyles and Fesler, 1990; Kane and Shuman, 1992; Rosel and Moss, 1985; Yang et al., 2011a). Intermediate and late genes are expressed after the onset of genome replication and are collectively termed post-replicative genes (Keck et al., 1990; Vos and Stunnenberg, 1988).

A distinct feature of poxviruses is their replication in cytoplasmic "viral factories" without direct involvement of the nucleus, which may engender a particular susceptibility to triggering, and the consequences of a type I interferon (IFN) response (Moss, 2013). In addition to encoding multiple proteins that inhibit the interferon regulatory factor 3 (IRF-3) and nuclear factor κB (NF-κB) signaling that would lead to expression of IFN-β (Smith et al., 2018), VACV encodes at least three proteins to directly evade type I IFN. Protein B18 is secreted from infected cells and binds to type I IFNs extracellularly, preventing their engagement with IFN receptors (Colamonici et al., 1995; Symons et al., 1995). Protein H1 dephosphorylates STAT molecules, blocking IFN signal transduction (Najarro et al., 2001). Protein C6 co-precipitates with STAT2 and inhibits transcription of IFN-stimulated genes (ISGs) (Stuart et al., 2016). C6 can also inhibit activation and nuclear translocation of IRF3, inhibiting IFN production (Unterholzner et al., 2011).

VACV also encodes proteins E3, K3, K1, C7, and C9 that are shown to, or are likely to, antagonize ISGs. Mechanisms of action identified include inhibition of activation of protein kinase R (PKR) or 2′5′-oligoadenylate synthetase (2′5′-OAS), inhibition of phosphorylation of eukaryotic initiation factor 2α to maintain viral

 © 2019 The Author(s).
This is an open access article under the CC BY license (http://creativecommons.org/licenses/by/4.0/).

protein synthesis, or inhibition of IRF1 (reviewed in Smith et al., 2018). However, only a single report has provided evidence of downregulation of an antiviral restriction factor by VACV. Interferon-induced transmembrane protein 3 (IFITM3) is downregulated during infection to evade antiviral restriction, although neither the viral mechanism of protein modulation, nor the cellular mechanism of VACV restriction by IFITM3, has been determined (Li et al., 2018).

A systematic quantitative analysis of temporal changes in host and viral proteins throughout the course of productive viral infection can provide dynamic insights into virus-host interaction. These include the prediction of components of innate and adaptive immunity as well as virus-host protein-protein interactions (Weekes et al., 2014). Furthermore, an unbiased analysis of proteins targeted by viruses for proteasomal degradation can predict antiviral factors (Nightingale et al., 2018). Using multiplexed tandem-mass tag (TMT)-based proteomics we measured ~9,000 host proteins and ~80% of VACV proteins over seven time points throughout infection, providing a comprehensive temporal view of the host proteome and VACV virome. Our analysis revealed that VACV downregulates 265 proteins, including multiple cell surface collagens and protocadherins, which may act as natural killer (NK) cell ligands. Other insights included the downregulation of multiple ISGs including all canonical IFN-induced proteins with tetratricopeptide repeats (IFITs). Studying infection in the presence or absence of the proteasomal inhibitor MG132 showed that 69% of downregulated proteins were targeted for proteasomal degradation, including histone deacetylase 5 (HDAC5). By generating a temporal system of classification of VACV protein expression, we predicted that the early viral protein C6 targets HDAC5 for degradation, which was confirmed using a mutant virus lacking C6 and a cell line expressing C6. Data presented define HDAC5 as a restriction factor that inhibits the replication of different families of DNA viruses.

RESULTS

Quantitative Temporal Viromic Analysis of Vaccinia Virus Infection

To build a global picture of changes in host and viral proteins throughout the course of VACV infection, we infected telomerase reverse transcriptase (TERT)-immortalized primary human fetal foreskin fibroblasts (HFFF-TERTs) with VACV strain Western Reserve (WR) at high multiplicity in biological triplicate. Flow cytometry confirmed that >95% of cells were infected (Figure S1A). Eleven-plex TMT and triple-stage mass spectrometry (MS3) were used to quantify changes in protein expression over seven time points (Figure 1A; Table S1). This quantified 8,991 human proteins and 172/216 viral proteins, providing a global view of changes in protein expression during infection (Figure S1B). Mock and early infection samples clustered separately from intermediate and late infection time points, with changes of the greatest magnitude occurring mostly late during infection (Figure 1B). Over 18 h of infection, 265 human proteins were downregulated >2-fold, and 70 human proteins upregulated >2-fold (Figures 1C and S1C; Tables S2A and S2B). This approach was validated by confirming the known downregulation of tumor suppressor protein p53 and upregulation of transcription factors Fos, Jun, and EGR1 (Figure S1D) (de Magalhães et al., 2001; Silva et al., 2006; Wali and Strayer, 1999; Yoo et al., 2008). Previously unreported findings included degradation of HDAC5 (Figure 1D). All data are shown in Table S1, in which the worksheet ''Plotter'' enables interactive generation of temporal graphs of the expression of each of the human or viral proteins quantified.

Cell Surface Proteins Targeted by Distinct DNA Viruses to Evade Immunity

The Database for Annotation, Visualization and Integrated Discovery (DAVID) software package (Huang da et al., 2009) was used to identify pathways enriched among proteins downregulated >2-fold. Interestingly, multiple clusters identified downregulated cell surface receptors and ligands, including the terms “extracellular,” “cell attachment site,” and “immunoglobulin-like fold” (Figure 2A; Table S2E). This suggests that regulation of plasma membrane proteins may be a key focus of VACV infection.

Poxviruses devote a considerable proportion of their coding capacity to manipulating host immunity (Burshtyn, 2013). Cowpox virus and myxoma virus downregulate surface major histocompatibility complex (MHC) class I (Guerin et al., 2002), VACV protein A40 resembles a C-type lectin and some NK cell receptors (Wilcock et al., 1999) and contributes to virulence (Tscharke et al., 2002), and VACV protein N1 may limit NK cell activity (Jacobs et al., 2008). However, there has been no systematic analysis of how VACV modulates NK or T cell recognition, in particular which ligands may be regulated in the infected cell. Therefore, these data were analyzed to determine which known NK and T cell ligands are down- or upregulated during infection. This revealed previously unrecognized modulation of multiple proteins. HLA-A, -B, and -C molecules were all downregulated during infection, in addition to nectin2, the ligand for activating NK receptor DNAM-1, and ULBP2, the ligand for activating NK receptor MHC class I polypeptide-related sequence A (MICA). The tumor necrosis factor (TNF) receptor superfamily member TNFRSF1A was also strongly downregulated (Figure S2A).

Most NK and T cell ligands belong to one of a few protein families, including immunoglobulins, C-type lectins, cadherins, TNF receptors, and major histocompatibility-complex-related molecules (Vivier et al., 2008). To identify candidate ligands that have not been recognized previously, we added InterPro functional domain annotations to our data (Hunter et al., 2012) and reasoned that modulation of a ligand during VACV infection may indicate biological importance. This showed that 37 proteins had a relevant InterPro annotation and were at least 2-fold downregulated compared to mock infection. Twelve collagens and four protocadherins were downregulated, in addition to the tyrosine protein kinase receptor AXL, endosialin (CD248), and numerous other molecules involved in adhesion, signaling, and immunity (Figures 2A–2C and S2B–S2D; Table S3).

Certain proteins important in immunity are targeted by more than one, or sometimes multiple, viruses (Schoggins et al., 2011; Schreiner and Wodrich, 2013). To identify proteins jointly downregulated by VACV and an unrelated double-stranded

OPEN ACCESS
CellPress

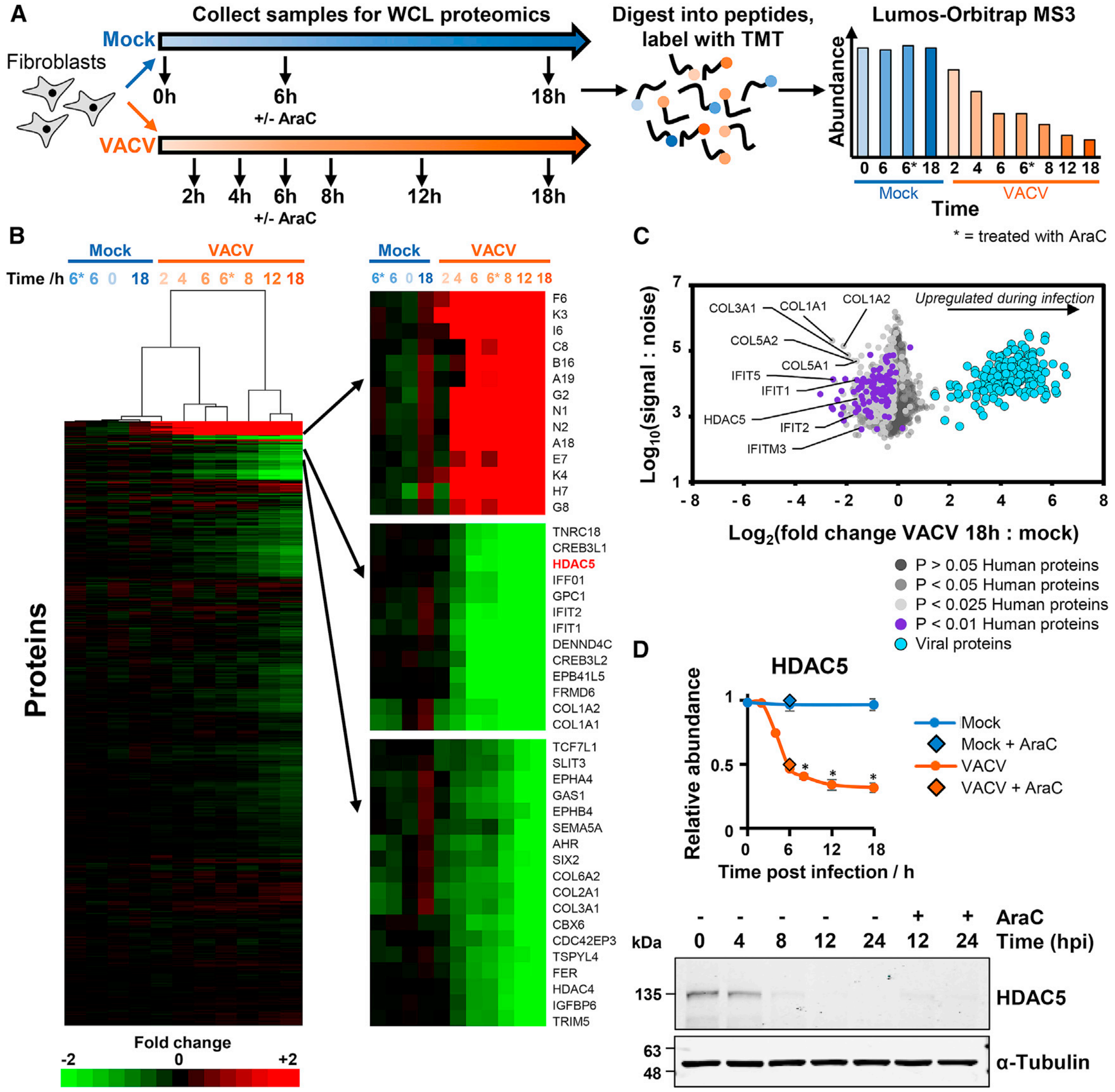

Figure 1. Quantitative Temporal Analysis of VACV Infection

(A) Schematic of experimental workflow for each of three biological replicates. Cells were infected at MOI of 5 or mock infected (Figure S1A). Additionally, one mock and one infected sample were treated for 6 h with the viral DNA replication inhibitor cytosine arabinoside (AraC).

(B) Hierarchical cluster analysis of all proteins quantified. An enlargement of three subclusters is shown (right panel), including multiple proteins that were substantially up- or downregulated.

(C) Scatterplot of all proteins quantified at 18 h of infection. For all analyses in this manuscript, a mean fold change at each time point was calculated by averaging fold changes from each of the biological replicates in which the protein was quantified. For the purposes of comparison, the 18-h mock sample from each replicate was used, because the 0-, 6-, and 18-h mock samples behaved extremely similarly (Figure S1E). To perform a comprehensive analysis, "sensitive" criteria were employed, examining proteins down- or upregulated >2-fold on average across all replicates in which the protein was quantified (Table S2A). Sensitive criteria were used in each analysis apart from where indicated. Data from "stringent" criteria that examined only proteins quantified in all three replicates with an average fold change > 2 and $p < 0.05$ are shown in Table S2C. For proteins quantified in all three replicates, a Benjamini-Hochberg-corrected two-tailed t test was used to estimate p values. Only proteins quantified in all three replicates are shown in this scatterplot.

(D) Example of a previously unreported target of VACV infection. Data are represented as mean ± SEM; *$p < 0.05$ (see STAR Methods). Immunoblot of HFFF-TERTs infected with VACV (MOI = 5) confirmed rapid HDAC5 downregulation.

DNA (dsDNA) virus, these data were combined with our previous quantitative temporal analysis of human cytomegalovirus (HCMV) infection (Figure 3A; Table S4A). The DAVID software suggested that proteins downregulated by both viruses were also enriched in cell surface receptors (Figure 3B; Tables S4B and S4C). Previously, we identified HCMV-induced downregulation of multiple protocadherins and provided initial evidence that members of this family are activating NK cell ligands (Weekes et al., 2014). Protocadherin γB5 was also downregulated during VACV infection, suggesting that this molecule might have a

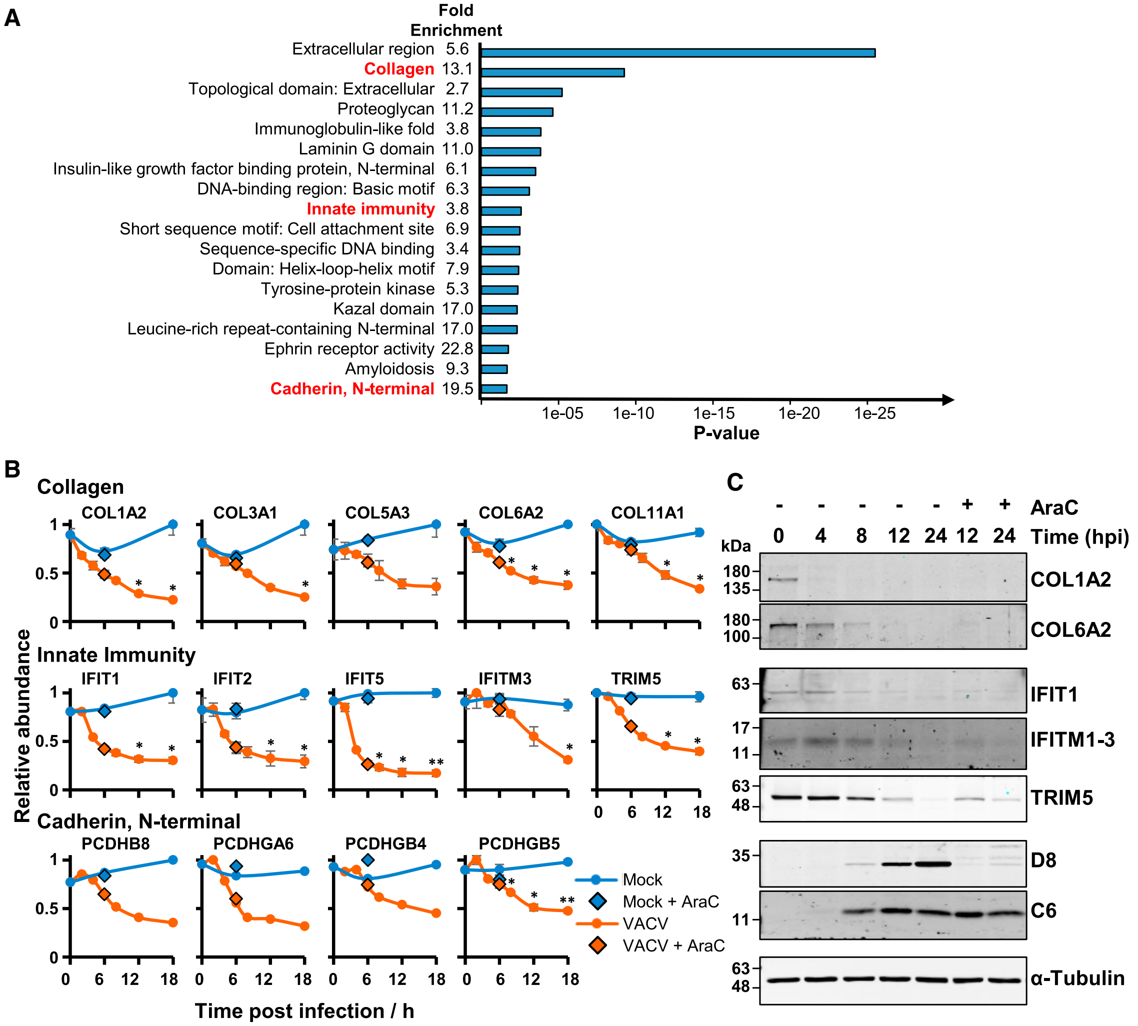

Figure 2. Downregulation of Multiple Collagens, Protocadherins, and Innate Immune Mediators

(A) Functional enrichment within all proteins that were downregulated >2-fold at any point during infection compared to 18-h mock samples. A background of all quantified human proteins was used. Shown are representative terms from each cluster with Benjamini-Hochberg-corrected p values of < 0.05. Components of each significantly enriched cluster are shown in Table S2E. A similar analysis was performed for proteins upregulated >2-fold; however, this did not reveal any significantly enriched clusters.

(B) Example temporal profiles of collagens, innate immune mediators, and protocadherins. Data are represented as mean ± SEM (n = 3); *p < 0.05 and **p < 0.01 (see STAR Methods). Error bars and statistics are not included on the plots for PCDHGA6, PCDHGB4, and PCDHB8, as these proteins were not quantified in all three replicates; full data are shown in Table S1.

(C) Validation of temporal profiles shown in (B) by immunoblot of HFFFs infected with VACV (MOI = 5). Viral proteins D8 and C6 were representative of late and early gene expression, respectively.

particularly important role in innate immunity (Figure 3C). Eleven collagens were downregulated by both viruses, and for two of these this was confirmed by immunoblot (Figure 2C). Certain collagens may be ligands for the inhibitory leukocyte-associated Ig-like receptor-1 (LAIR-1) (Lebbink et al., 2008), suggesting that their downregulation may be consistent with an appropriate response to intracellular infection. Given that certain NK ligands can have distinct roles depending on which receptor they bind (Vivier et al., 2008), such broad collagen downregulation might equally be specifically triggered by VACV to engender immune evasion.

Downregulation of Antiviral Factors and Interferon-Stimulated Genes

Enrichment analysis revealed marked downregulation of proteins in the category “innate immunity” (Figure 2B; Table S2E). This included IFITM3, which has recently been reported to be downregulated by VACV to evade IFITM3-mediated antiviral restriction (Li et al., 2018). Strikingly, multiple IFITs were also downregulated; a comprehensive search revealed that all four canonical IFITs 1, 2, 3 and 5 were rapidly downregulated during infection (Table S2A). The IFITs are induced upon stimulation with IFN or viral infection, have homologs in multiple vertebrate

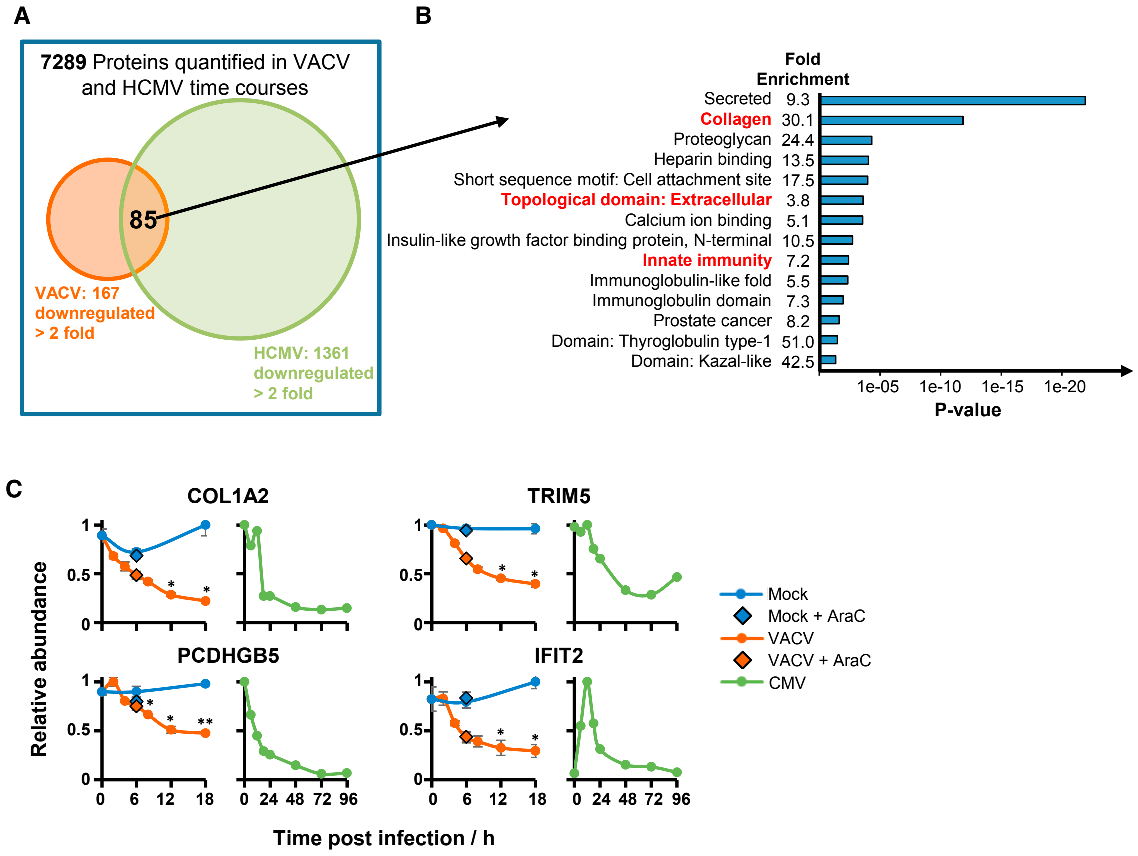

Figure 3. Co-regulation of Proteins by VACV and HCMV

(A) Overlap between VACV and HCMV whole cell lysate proteomic data (Weekes et al., 2014).

(B) DAVID enrichment analysis of 85 co-regulated proteins against a background of all 7,289 proteins quantified in both studies.

(C) Example temporal profiles of proteins from enriched clusters. For VACV data, data are represented as mean ± SEM (n = 3); *p < 0.05 and **p < 0.01 (see STAR Methods).

species, and play key roles in restricting a diversity of RNA viruses including Rift Valley fever virus (RVFV), vesicular stomatitis virus (VSV), and influenza A (Vladimer et al., 2014). The canonical mechanism of IFIT restriction is thought to be recognition of RNA modified via 5′ triphosphorylation, or lacking methylation at the 2′-*O* of the 5′ guanosine. Direct inhibition of RNA viral translation has also been reported (Daffis et al., 2010; Pichlmair et al., 2011). Our observation that both VACV and HCMV downregulate IFITs (Figure 3C; Table S4C) suggests that this whole class of proteins may have an as yet unrecognized mechanism of restricting DNA viruses in addition to RNA viruses.

We quantified 29 tripartite motif containing proteins (TRIMs), of which TRIM 5, 13, 25, 26, and 56 were downregulated during infection. TRIM5 was also targeted by HCMV (Figure 3C; Tables S2A and S2B). TRIM5 can restrict retroviruses, and TRIM56 inhibits diverse RNA viruses including influenza, dengue, and yellow fever virus (Liu et al., 2014, 2016; Rahm and Telenti, 2012). Interestingly, TRIM56 has recently also been shown to monoubiquitylate the cytosolic sensor cyclic GMP-AMP (cGAMP) synthase (cGAS), resulting in a marked increase in cGAMP production. Mice deficient in TRIM56 exhibited increased susceptibility to lethal infection by herpes simplex virus type 1 (HSV-1) (Seo et al., 2018). It is therefore possible that downregulation of TRIM56 by VACV represents another mechanism of viral evasion of DNA sensing pathways, and suggests that further examination of the IFITs, IFITMs, and TRIMs may identify DNA viral restriction factors, or components of antiviral pathways.

Temporal Analysis of Vaccinia Viral Protein Expression

Recent studies of temporal VACV gene expression have employed transcriptional approaches including microarrays (Assarsson et al., 2008), RNA sequencing (RNA-seq) (Yang et al., 2010, 2011b), and most recently a combination of mRNA-seq and ribosomal profiling (Yang et al., 2015). These have enabled an updated classification of viral transcripts, including the definition of early transcript classes E1.1 and E1.2 (Yang et al., 2010, 2011b). Furthermore, Croft et al. (2015) infected a murine bone marrow-derived dendritic-like cell line DC2.4 to quantify 101 VACV proteins over two independent time courses up to 9 h after infection, classifying four protein expression clusters.

A high-definition temporal study of viral protein expression over the whole course of viral infection has the potential to

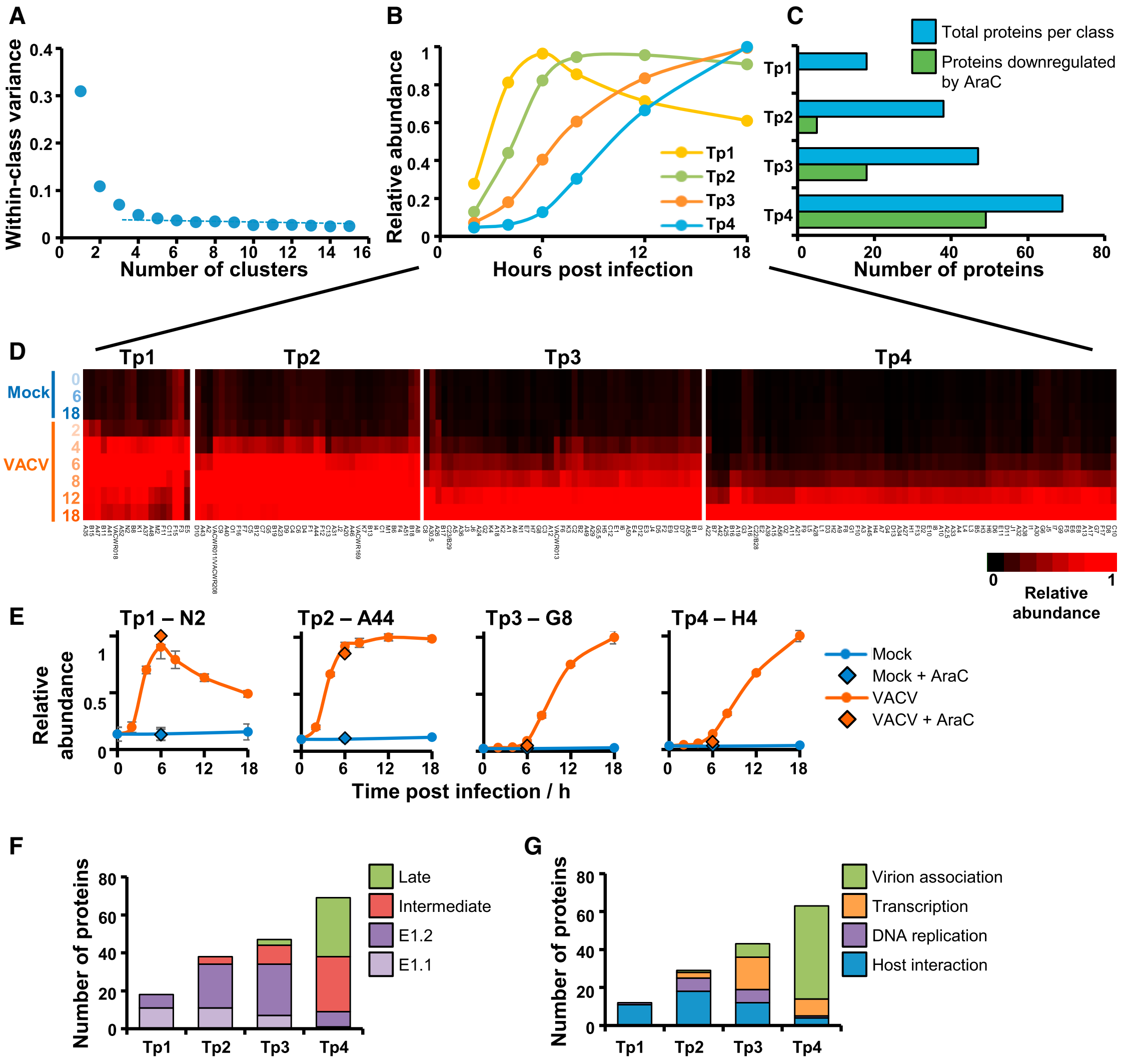

Figure 4. Definition of Temporal Classes of VACV Gene Expression

(A) Number of temporal classes of VACV gene expression. The k-means approach was used with 1–15 classes to cluster viral proteins, and the summed distance of each protein from its cluster centroid was calculated. Although this summed distance necessarily becomes smaller as more clusters are added, the rate of decline decreases with each added group, eventually settling at a fairly constant rate of decline that reflects overfitting; clusters added prior to this point reflect the underlying structure in the temporal protein data, whereas clusters subsequently added through overfitting are not informative. The point of inflexion fell between four and six classes, suggesting that there are at least four distinct temporal protein profiles of viral protein expression.

(B) Class centroid profiles.

(C) Number of viral proteins per class, and the number of proteins in each class whose expression was reduced >1.5-fold by incubation with AraC (treated and untreated samples both assessed at 6 h of infection; see schematic in Figure 1A and results in Figure S3).

(D) Temporal profiles of proteins in each k-means class were subjected to hierarchical clustering by Euclidian distance.

(E) Temporal profiles of representative proteins from each cluster. Data are represented as mean ± SEM (n = 3).

(F) Comparison of viral protein and transcript classes.

(G) Functional analysis of viral proteins, based on information from Yang et al. (2010) and other references detailed in Table S5.

provide a complementary system of protein classification, in addition to enabling direct correlation between viral and cellular protein profiles to give insights into viral-host protein interaction (Weekes et al., 2014). We quantified ~80% of all predicted VACV proteins. The number of classes of viral protein expression was determined by clustering viral proteins using the k-means method, which suggested that there are at least four distinct temporal protein profiles of viral protein expression (Figures 4A–4C; proteins of each class are shown in Figures 4D and 4E and Table S5).

To directly compare transcriptional and protein temporal classes, viral protein expression was examined first in the presence of cytosine arabinoside (AraC). None of the proteins in the earliest temporal class (temporal profile 1, Tp1) were inhibited by AraC, compared to >70% of proteins in the latest class (Tp4). Proteins that were not apparently inhibited by AraC in

the latest class may simply reflect the stringent threshold employed, or the time point of harvesting of AraC-treated samples. We next compared our protein-level data with an RNA-seq-based definition of transcriptional classes from the manuscripts of Yang et al. (2010, 2011b). Comparison to the protein data was striking: 18/18 Tp1 proteins were E1.1 or E1.2 class transcripts, and 60/69 Tp4 proteins were I or L transcripts ($p < 0.0001$, Fisher's exact test). The Tp2 class of proteins was additionally enriched in E1.2 transcripts (Figures 4F and S4A; Table S5). Such correspondence between different studies targeting distinct classes of biomolecules suggests that the temporal classes of VACV protein expression defined here are likely to be biologically relevant, and suggests that the temporal regulation of VACV gene expression is chiefly exerted at the transcriptional level. The comparison also highlights some interesting differences. For example, certain transcripts produced early during infection were not expressed maximally until later as Tp3-class proteins, including DNA-dependent RNA polymerase subunits E4 and G5.5, and G2, which has roles in post-replicative transcription elongation (Figure S4A, cluster 3). This suggests that additional mechanisms for temporal regulation of virus gene expression might operate during infection.

Additionally, our data were compared to 47 viral proteins that were assigned the same temporal class in each of two time courses by Croft et al. (2015). These proteins exhibited similar classifications between both studies: 4/5 Tp1 proteins were Croft et al. temporal class 1 and 19/20 Tp4 proteins were Croft et al. temporal classes 3 or 4 (Figure S4B; Table S5).

To further compare protein function with temporal class, we annotated our viral protein data with functions derived from a summary of multiple literature sources (Table S5). The category "virion association," which included virion structural proteins or proteins involved in virion morphogenesis chiefly related to the Tp4 class, while "DNA replication" (including enzymes involved in nucleotide precursor synthesis, DNA replication, and DNA processing) related to Tp2 proteins. Proteins functioning in "host interaction" (including the vaccinia growth factor [VGF] and immune evasion proteins) were mainly expressed early (Figure 4G).

Systematic Analysis of Protein Degradation during VACV Infection

Previously, we described a multiplexed approach to discover proteins with innate immune function on the basis of active degradation by the proteasome during HCMV infection (Nightingale et al., 2018). This approach was adapted to determine which of the proteins that are downregulated by VACV are also proteasomally degraded. MG132 or DMSO was added 2 h after VACV or mock infection to enable virus uncoating in the host cells prior to inhibition of the proteasome. An MG132 "rescue ratio" for each of 8,263 quantified proteins was obtained by comparing protein abundance during VACV infection ± inhibitor with protein abundance during mock infection ± inhibitor. This ratio enabled identification of proteins that exhibited increased degradation during VACV infection, as opposed to those having a high baseline turnover in mock-infected cells. Of the proteins downregulated >2-fold, 69% had a rescue ratio >1.5 with $p < 0.05$, suggesting that one of the predominant mechanisms VACV employs to downregulate proteins is proteasomal degradation (Figure 5A). All IFITs, TRIMs, and Ephrin receptors downregulated >2-fold were rescued by MG132. By contrast, only COL6A2 proteins (isoforms 1 and 2) were rescued; six other downregulated collagens did not meet the criteria for rescue, suggesting an alternative mechanism of downregulation (Figures 5A and 5B; Table S6A).

Consistent with previous reports, MG132 inhibited late gene expression, but not expression of early genes (Satheshkumar et al., 2009; Teale et al., 2009). Furthermore, dividing viral proteins into temporal classes revealed that most Tp1 and some Tp2 proteins were upregulated by MG132; Tp3 class proteins were largely unaffected, and Tp4-class proteins were inhibited (Figure 5C). This suggests that many of the earliest-expressed VACV proteins may be proteasomally degraded. Given that Tp1 and Tp2 class proteins are enriched in "host interaction" functions (Figure 4G), this may partly be explained by co-degradation of some of these viral proteins with their host targets. Otherwise, the upregulation of some Tp1 and Tp2 proteins might be a consequence of the inhibition of viral DNA replication and post-replicative gene expression by MG132, leading to a prolonged accumulation of early viral mRNAs and their protein products (Baldick and Moss, 1993; Parrish and Moss, 2006)

Identification of Candidate Viral-Host Interactions

VACV has a dsDNA genome of 191 kbp and is predicted to encode >200 proteins (Goebel et al., 1990). Identification of which viral protein targets a given cellular factor can therefore be a challenging task. We recently described an approach that makes direct comparison between viral and cellular protein profiles (Weekes et al., 2014). HDAC5 was rapidly degraded during infection (Figure 1D), was one of the proteins most substantially rescued by MG132 (Figures 5A and 6A), and was also downregulated by HCMV, suggesting that this molecule might be particularly important in the life cycle of diverse viruses (Tables S2A and S4B). To determine which class of viral protein targets HDAC5, we compared the four protein class centroid profiles (Figure 4B) to an inverted profile of HDAC5. This most closely matched Tp2 class proteins (Figure 6B), suggesting that one of the 38 proteins in this class may target HDAC5.

HDAC5 Is Targeted by the VACV C6 Protein

To determine which viral protein targets HDAC5, we compared the profiles of the Tp2-class viral proteins known to have roles in innate immune signaling to the profile of HDAC5. Using Euclidian distance as a measure of difference between protein profiles, the expression kinetics of protein C6 most closely matched the kinetics of HDAC5 downregulation (Figure 6C). An unbiased proteomic comparison of HFFF-TERTs infected with wild-type (WT) VACV or a mutant lacking the *C6L* gene (Unterholzner et al., 2011), vΔC6, showed that HDAC5 was targeted by C6 (Figure 6D; Table S1). The specific regulation of this HDAC was confirmed by a lack of any effect of infection with either virus on HDAC1 (Figures 6A and 6E; Table S1).

The proteomic findings that the ubiquitin-proteasome system is co-opted by VACV to degrade HDAC5 (Figure 6A) was confirmed by immunoblot analysis. MG132 reduced late gene expression, represented by protein D8, but not expression of

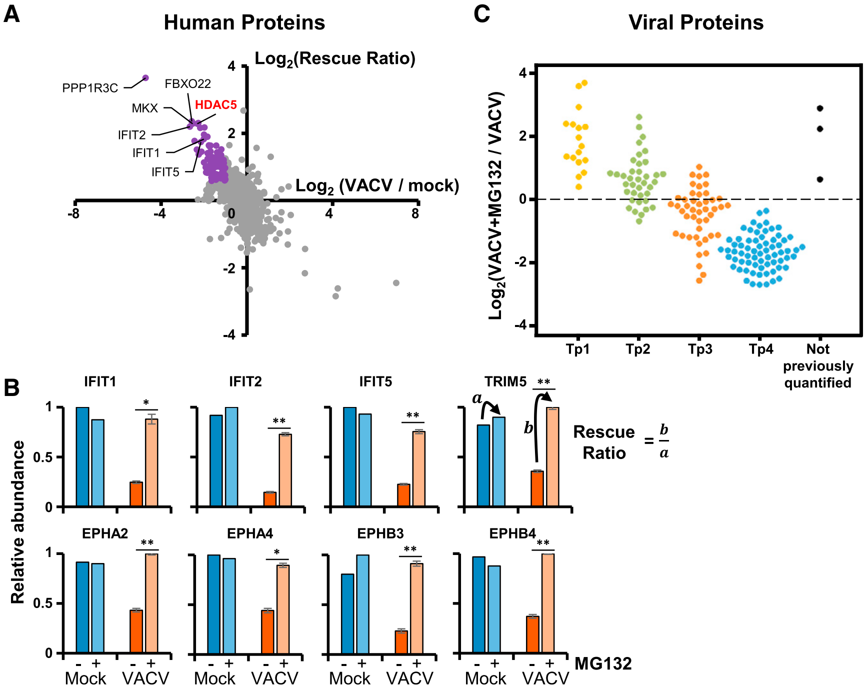

Figure 5. Systematic Analysis of Proteasomal Degradation

(A) Scatterplot to identify human proteins that are both downregulated during VACV infection and rescued by proteasomal inhibition. Cells were infected at MOI 5 for 12 h in biological triplicate. MG132 or DMSO control was added 2 h after infection.

(B) A "rescue ratio" was calculated: (protein abundance during VACV infection with MG1322 / abundance during infection without MG132) (b) / (protein abundance during mock infection with MG132 / abundance without MG132) (a). Here, (a) was limited to a minimum of 1 to avoid artificial ratio inflation. To fit the constraints of an 11-plex TMT experiment, mock samples (± MG132) were examined in single replicates (Table S7B). For each protein, a Benjamini-Hochberg-corrected two-tailed t test was used to estimate a p value where the infected samples with MG132 were different from the infected samples without MG132. Proteins that exhibited >2-fold downregulation during infection, a rescue ratio >1.5-fold, and $p < 0.05$ are colored purple. Data underlying this figure are shown in Table S6A.

(B) Example data for host proteins that were rescued by proteasomal inhibition. The p values for virally infected biological triplicates were calculated as described in (A). For infected samples, data are represented as mean ± SEM; *$p < 0.001$ and **$p < 0.0005$ (see STAR Methods).

(C) Regulation of viral proteins by proteasome inhibition. Data underlying this figure are shown in Table S6B. Three proteins were quantified in this experiment, but not in any of the three time course replicates, and are indicated in black.

early protein C6 (Satheshkumar et al., 2009; Teale et al., 2009), and inhibited HDAC5 degradation (Figure 6F). Furthermore, HDAC5 downregulation was also observed in the presence of AraC at 6, 12, and 24 h, indicating that an early gene was necessary and intermediate and late gene expression were not needed (Figure 1D). C6 was also shown to be sufficient for HDAC5 degradation by its inducible expression in HEK.TetR cells (Figure 6G).

HDAC5 Restricts VACV and HSV-1 Infection

The rapid downregulation of HDAC5 by VACV early during infection, and downregulation by a distinct large DNA virus, HCMV, suggested a role in antiviral restriction. This was confirmed by the observation that inducible overexpression of HDAC5 in U2OS cells restricted replication of both VACV and HSV-1 (Figures 7A and 7B). Further, in four independently derived CRISPR/Cas9 knockout clones (in HeLa and HEK293T cells), the replication of both viruses was enhanced compared to control (Figures 7C–7H). Re-introduction of HDAC5 restored restriction of these viruses (Figure 7E). Collectively, these data show that HDAC5 is a restriction factor for two different large DNA viruses, and the biological importance of this is supported by the targeted degradation of HDAC5 during VACV and HCMV infection.

DISCUSSION

Recent advances in quantitative proteomics have provided the opportunity to deepen our understanding of variations in the cellular proteome under physiological or pathological conditions. Until now there have been only two proteomic analyses of host and viral proteins during VACV infection, both limited by the technology available to the investigators at the time. In the first, only 24 human and 3 viral proteins were quantified due to the use of two-dimensional gel electrophoresis to pick individual protein spots for analysis (Bartel et al., 2011). In the second, a total of ~3,400 proteins were quantified at a single late time after infection. However, nearly all of the proteins that changed in response to infection were viral gene products, with very little modulation of the host proteome observed (Chou et al., 2012). In this study, we applied quantitative temporal viromics (Weekes et al., 2014) to take a comprehensive view of the changes in the viral and cellular proteomes throughout VACV infection. We labeled peptides with TMT tags and measured proteins using MS3 on an Orbitrap Lumos, which can provide a uniquely precise quantitative measurement of protein abundances on a near-global proteomic scale (Ting et al., 2011). Our data thus provide a valuable resource for future studies of poxvirus infection.

To replicate in the cytoplasm, poxviruses have developed multiple strategies to modulate intrinsic host defenses. One benefit is that VACV infection can be used to discover facets of host immunity, because viral modulation of the host often reflects biological importance. Multiple members of certain protein families were regulated during infection, suggesting that these proteins may play a particularly important role in host defense. For example, multiple collagens and protocadherins were downregulated, some of which may be NK ligands.

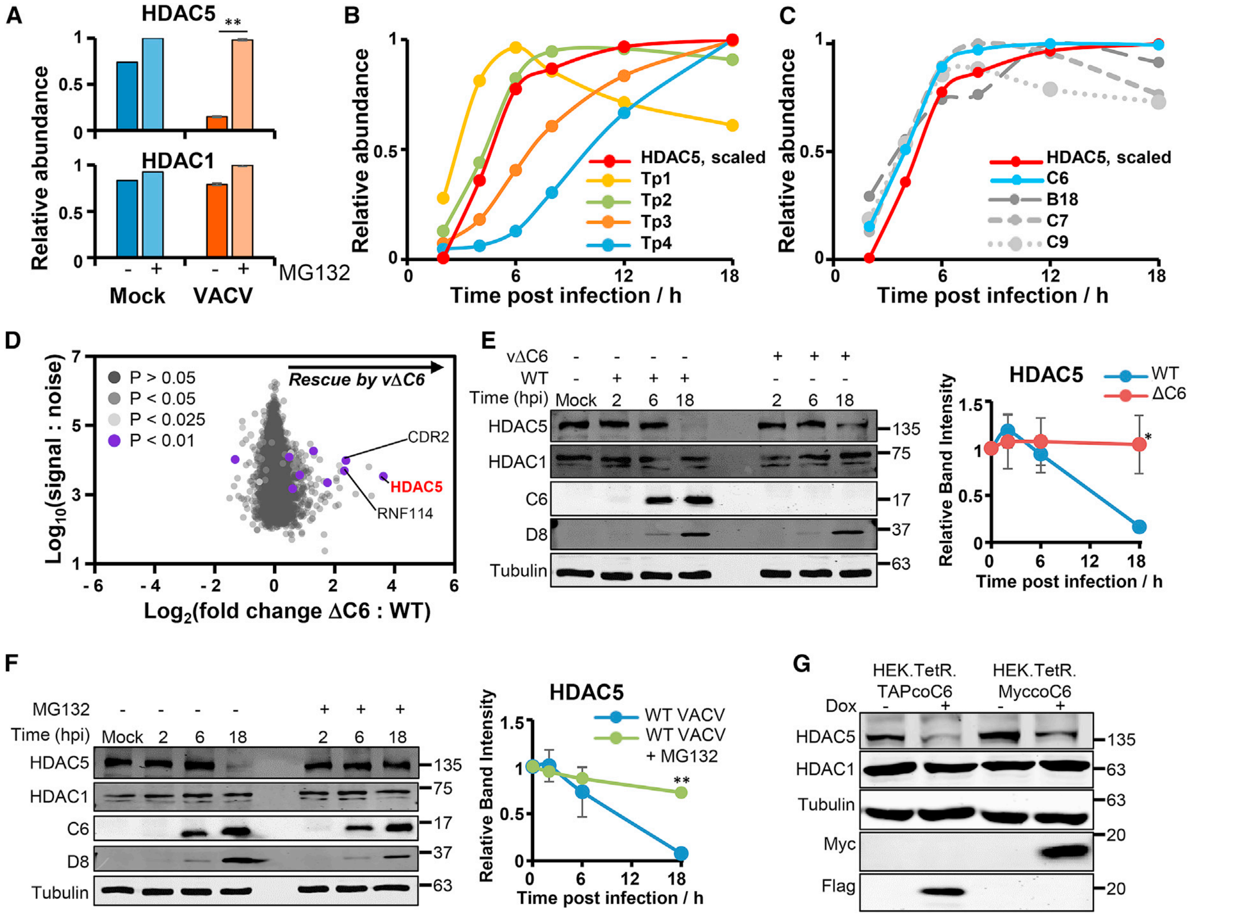

Figure 6. VACV Protein C6 Downregulates HDAC5

(A) HDAC5, but not HDAC1, is proteasomally degraded during VACV infection. Bar charts and statistics were generated as described in Figure 5.

(B) Viral class centroid profiles compared to an inverted profile of HDAC5, which had additionally been scaled from 0 to 1.

(C) Profile of HDAC5 scaled as in (B), and Tp2-class VACV proteins with known roles in regulation of IFN or ISGs.

(D) C6 targets HDAC5. HFFF-TERTs were infected in biological triplicate with WT VACV or vΔC6 (lacking gene *C6L*) (Unterholzner et al., 2011) (MOI = 5 and 12 h). The scatterplot shows all proteins quantified. A Benjamini-Hochberg-corrected two-tailed t test was used to estimate p values.

(E) Representative immunoblot demonstrating that C6 specifically targets HDAC5. By comparison, HDAC1 was unmodified (MOI = 5). Quantitation of all three replicate immunoblots are shown (right panel). Data are represented as mean ± SEM, *p < 0.05 from a two-tailed t test.

(F) Representative immunoblot demonstrating rescue of HDAC5 expression by inhibition of the proteasome (MOI = 5). MG132 was added 2 h after VACV infection. Quantitation of all three replicate immunoblots is shown (right panel). Data are represented as mean ± SEM, **p < 0.00005 from a two-tailed t test.

(G) Immunoblot demonstrating that inducible expression of C6 is sufficient for HDAC5 degradation. HEK293T cells inducibly expressing C6 with an N-terminal Myc or C-terminal TAP tag were either treated or untreated with 100 ng/ml doxycycline overnight then lysates immunoblotted.

Nevertheless, only 265/8,991 proteins were downregulated >2-fold even during late infection, despite the profound shutdown of host protein synthesis induced by the virus (Moss, 1968; Parrish and Moss, 2007; Parrish et al., 2007; Rice and Roberts, 1983; Strnadova et al., 2015). This is in contrast to our previous study of HCMV infection (which does not cause host shutoff), in which 1,740/7,491 proteins were downregulated >2-fold (Weekes et al., 2014). HCMV and VACV have a broadly similar number of canonical genes. The differential regulation of the host observed might therefore reflect differences in viral biology. We have shown that certain individual herpesviral proteins can regulate multiple host targets. For example, HCMV US2 can alone regulate ≥ 21 proteins including MHC molecules and integrins, and a single Kaposi's sarcoma herpesvirus protein, K5, can regulate 83 cell surface proteins (Hsu et al., 2015; Timms et al., 2013). It is possible that VACV proteins each individually have a paucity of discrete functions; thus, the summed effect of ~200 viral genes is relatively modest. Nevertheless, C6 had at least 3 targets including E3 ubiquitin ligase RNF114 and Cerebellar degeneration-related protein 2 (CDR2) (Figure 6D). Alternatively, the degree of regulation of the host by VACV may be small in comparison to HCMV. Since all herpesviruses exhibit latent as well as lytic infection, and must enter and exit the nucleus during lytic replication, such increased complexity to the viral life cycle may also require increased regulation of the infected cell.

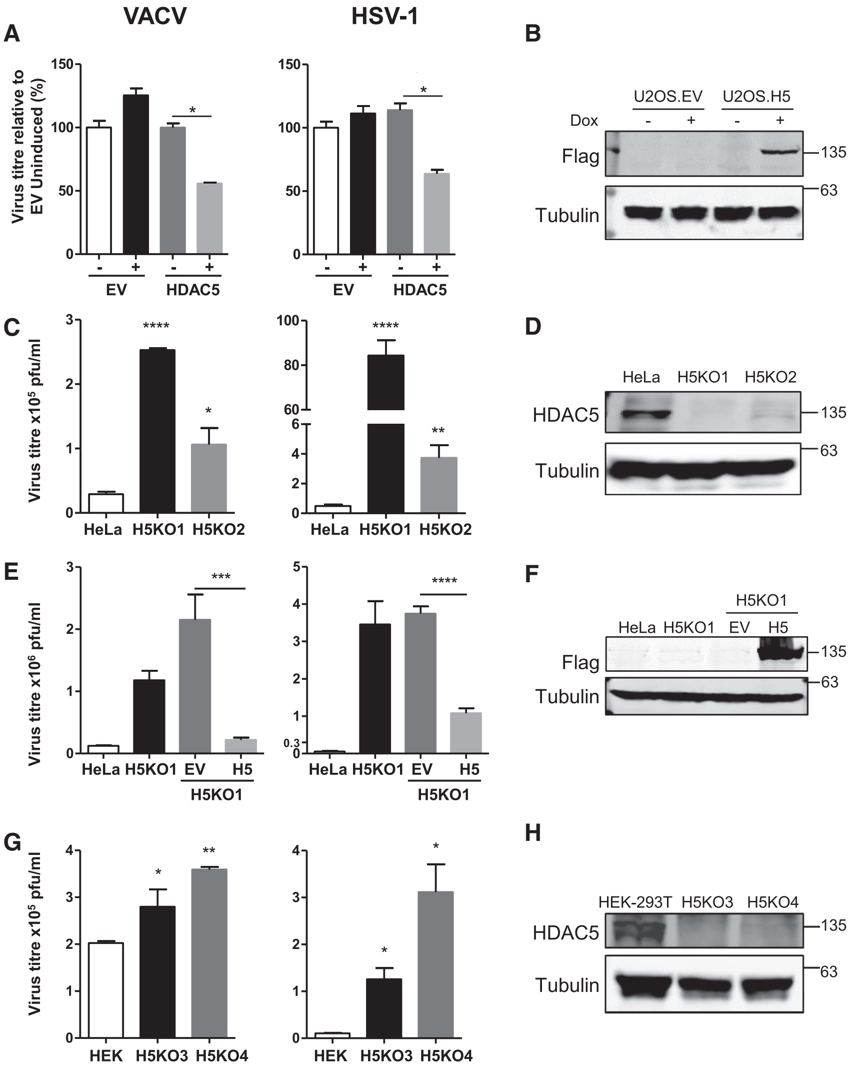

Figure 7. HDAC5 Restricts VACV and HSV-1 Infection

(A) Diminished replication of VACV and HSV-1 in an U2OS cell line inducibly expressing HDAC5-FLAG. Infections were performed at MOI = 0.001, for 2 days with VACV and 3 days with HSV-1, after induction with 100 ng/ml doxycycline overnight. Data are represented as mean ± SEM, p values were calculated using a two-tailed t test (n = 3). *p < 0.05 and **p < 0.01; ns: not significant.

(B) Immunoblot of HDAC5-FLAG expression in U2OS cell lines.

(C) Enhanced replication of VACV and HSV-1 in HeLa CRISPR/Cas9 HDAC5$^{-/-}$ knockout clones compared to parental cell lines. Infections were performed as detailed in (A). Data are represented as mean ± SEM, p values were calculated using a two-tailed t test (n = 3). *p < 0.05, **p < 0.01, and ***p < 0.001.

(D) Immunoblot confirmed knockout of HDAC5. Sequencing of genomic DNA from clones H5KO1 and H5KO2 confirmed frameshift mutations in both alleles (Table S7A).

(E) Reintroduction of HDAC5 in HDAC5$^{-/-}$ cells restored restriction of VACV and HSV-1 replication. H5KO1 cells were transduced with either empty vector (EV) or HDAC5-FLAG. Infections were performed as detailed in (A). Data are represented as mean ± SEM, p values were calculated using a two-tailed t test (n = 3). ***p < 0.001 and ****p < 0.0001.

(F) Immunoblot analysis of HDAC5-FLAG expression in H5KO1 cell lines.

(G) Enhanced replication of VACV and HSV-1 in HEK293T CRISPR/Cas9 HDAC5$^{-/-}$ knockout clones compared to parental cell lines. Infections were performed as detailed in (A). Data are represented as mean ± SEM, p values were calculated using a two-tailed t test (n = 3). *p < 0.05 and **p < 0.01.

(H) Immunoblot confirming knockout of HDAC5. Sequencing of genomic DNA from clones H5KO3 and H5KO4 confirmed frameshift mutations in both alleles (Table S7A).

One benefit of quantifying common protein changes induced by distinct classes of viruses is the prediction of molecules of particular importance in immunity. The finding that HDAC5 is downregulated by both HCMV and VACV suggested that this molecule might play an important role during infection, such as in antiviral restriction. We demonstrate that HDAC5 indeed restricts both VACV and HSV-1. Previously, HDAC5 was reported to interact with proteins from other viruses, although had not hitherto been identified as a restriction factor, or shown to be downregulated during infection. The HSV-1 immediate protein ICP0 interacted with HDAC5 and inhibited its repression of the cellular myocyte enhancer factor 2 (MEF2) promoter (Lomonte et al., 2004). HDAC5 could repress activation of the Epstein-Barr virus (EBV) latent membrane protein 1 (LMP1) promoter by the EBV protein EBNA2. Repression was overcome by the co-activator EBNA-LP (Portal et al., 2006). Potential mechanisms that could explain restriction by HDAC5 of viruses replicating in distinct cellular niches therefore include inhibition of viral promoters (via HDAC5 nucleocytoplasmic shuttling) or, possibly, modulation of the IFN pathway or other innate immune signaling pathways. This is under investigation in our laboratory. The VACV C6 protein regulates both IRF-3 and type I IFN signaling and co-precipitates with the TBK1 adaptor proteins SINTBAD, NAP1, and TANK (Unterholzner et al., 2011), and with STAT2 via the transactivation domain (TAD) (Stuart et al., 2016). HDAC5 is also known to co-precipitate with STAT2 (Nusinzon and Horvath, 2003). Future studies will address if and how HDAC5 is ubiquitinated, and how C6 interacts with HDAC5, E2, and E3 ligases and any other required accessory proteins.

Mechanisms for protein downregulation by VACV include the well-described host shutoff via viral decapping enzymes and XRN1 mediated degradation of host transcripts (Burgess and Mohr, 2015; Liu et al., 2015; Parrish and Moss, 2007; Parrish et al., 2007; Rice and Roberts, 1983; Yang et al., 2010). However, given that the great majority of cellular proteins did not decrease in abundance, a general reduction in mRNA levels, or translation of these, was unlikely to be the major cause of the 265 proteins

being reduced more than two fold. Indeed, we found that MG132 rescues 69% of host proteins from degradation including HDAC5, suggesting that many of the downregulated proteins may be specifically targeted to the proteasome. This finding is consistent with a recent analysis of protein ubiquitylation during cowpox virus infection (Grossegesse et al., 2018). Transcriptional mechanisms may nevertheless play an important role in protein downregulation. For example, all collagens downregulated >2-fold by VACV apart from COL6A2 were not degraded in the proteasome. In our previous analysis of protein degradation during HCMV infection, 10/11 collagens downregulated >2-fold were transcriptionally, and not proteasomally regulated (Nightingale et al., 2018). This suggests either that both viruses regulate this class of molecules similarly or that transcriptional repression of collagen expression is a cellular response to infection.

In addition to HDAC5, we found that all IFITs and certain TRIMs were rapidly degraded during infection. IFIT1 mRNA and protein was very recently demonstrated to be downregulated by 24 h of VACV infection, in part via mammalian target of rapamycin (mTOR)-dependent cGAS degradation (Meade et al., 2018). Restriction of VACV by IFITs and TRIMs would highlight additional viral strategies to usurp IFN-mediated antiviral effectors. Candidate viral effectors include the Tp2-class C9 protein, which was recently mapped as an antagonist of IFN action and shown to co-precipitate with components of the SCF (SKP, cullin-1, F-box) E3 ligase complex (Liu and Moss, 2018). Host targets that may be degraded by C9 are not yet described; however, of note is that C9 itself was significantly upregulated by MG132 (Table S1), which may suggest it is co-degraded (Nightingale et al., 2018). In addition to viral uncoating, the proteasome has been shown to be required for VACV DNA replication; however, it had hitherto been unclear why (Mercer et al., 2012; Satheshkumar et al., 2009; Teale et al., 2009). A possible explanation may be to overcome HDAC5 restriction and to permit virus infection to progress beyond genome replication. Alternatively, proteasome activity may be necessary to directly regulate viral or cellular proteins involved in replicating viral DNA in cytoplasmic viral factories.

The temporal quantitation of ~80% of predicted VACV proteins in a single experiment provides a substantial advance. Understanding of VACV gene expression had hitherto mainly been at the transcript level. We now provide a temporal system of classification of VACV protein expression complementary to and consistent with the transcriptionally based E1.1/E1.2/I/L nomenclature. A particular use of these temporal protein profiles is the ability to correlate viral and host protein expression, which can predict the class of viral proteins responsible for modulation of each host protein. With additional functional information, such as a knowledge of which proteins in each class can modulate IFN, the individual viral gene required can readily be identified, as shown here for the degradation of HDAC5 by C6.

Using a powerful multiplexed proteomics approach, we have therefore identified VACV cellular targets, and defined protein C6, a multifunctional IFN antagonist, as a factor inducing proteasomal degradation of multiple host restriction factors. In addition to inhibiting IRF-3 activation and type I IFN-induced signaling, C6-induced degradation of HDAC5 joins the increasing panoply of functions encoded by VACV to evade viral restriction.

STAR★METHODS

Detailed methods are provided in the online version of this paper and include the following:

- KEY RESOURCES TABLE
- CONTACT FOR REAGENT AND RESOURCE SHARING
- EXPERIMENTAL MODEL AND SUBJECT DETAILS
  - Cells and Cell Culture
  - Viruses
  - Plasmids
- METHOD DETAILS
  - Virus Infections and Inhibitors
  - Lentiviral Transduction
  - CRISPR/Cas9-Mediated Gene Knockout
  - Immunoblotting
  - Flow Cytometry
  - Whole Cell Lysate Protein Digestion
  - Peptide Labeling with Tandem Mass Tags
  - Offline HpRP Fractionation
  - LC-MS3
- QUANTIFICATION AND STATISTICAL ANALYSIS
  - Data Analysis
  - Statistical Analysis
  - Pathway Analysis
- DATA AND SOFTWARE AVAILABILITY

SUPPLEMENTAL INFORMATION

Supplemental Information can be found online at https://doi.org/10.1016/j.celrep.2019.04.042.

ACKNOWLEDGMENTS

We are grateful to Prof. Steve Gygi for providing access to the "MassPike" software pipeline for quantitative proteomics and to Mrs. Rachel Seear for technical support. This work was supported by a Wellcome Trust Principal Research Fellowship (090315) to G.L.S., a Wellcome Trust Senior Clinical Research Fellowship (108070/Z/15/Z) to M.P.W., and a strategic award to Cambridge Institute for Medical Research, from the Wellcome Trust (100140). This study was additionally supported by the Cambridge Biomedical Research Centre, UK.

AUTHOR CONTRIBUTIONS

L.S., J.D.A., Y.L., M.P.W., and G.L.S. designed the experiments. L.S., J.D.A., Y.L., M.P.W., and G.L.S. wrote the manuscript. L.S., J.D.A., Y.L., C.T.R.D., and R.A. performed the experiments. L.S. and M.P.W. analyzed the proteomics data. L.S., J.D.A., Y.L., R.A., M.P.W., and G.L.S. edited the manuscript. M.P.W. and G.L.S. supervised all research.

DECLARATION OF INTERESTS

The authors declare no competing interests.

Received: September 11, 2018
Revised: February 21, 2019
Accepted: April 8, 2019
Published: May 7, 2019

REFERENCES

Assarsson, E., Greenbaum, J.A., Sundström, M., Schaffer, L., Hammond, J.A., Pasquetto, V., Oseroff, C., Hendrickson, R.C., Lefkowitz, E.J., Tscharke, D.C., et al. (2008). Kinetic analysis of a complete poxvirus transcriptome reveals an immediate-early class of genes. Proc. Natl. Acad. Sci. USA *105*, 2140–2145.

Baldick, C.J., Jr., and Moss, B. (1993). Characterization and temporal regulation of mRNAs encoded by vaccinia virus intermediate-stage genes. J. Virol. *67*, 3515–3527.

Bartel, S., Doellinger, J., Darsow, K., Bourquain, D., Buchholz, R., Nitsche, A., and Lange, H.A. (2011). Proteome analysis of vaccinia virus IHD-W-infected HEK 293 cells with 2-dimensional gel electrophoresis and MALDI-PSD-TOF MS of on solid phase support N-terminally sulfonated peptides. Virol. J. *8*, 380.

Broyles, S.S., and Fesler, B.S. (1990). Vaccinia virus gene encoding a component of the viral early transcription factor. J. Virol. *64*, 1523–1529.

Burgess, H.M., and Mohr, I. (2015). Cellular 5′-3′ mRNA exonuclease Xrn1 controls double-stranded RNA accumulation and anti-viral responses. Cell Host Microbe *17*, 332–344.

Burshtyn, D.N. (2013). NK cells and poxvirus infection. Front. Immunol. *4*, 7.

Carter, G.C., Rodger, G., Murphy, B.J., Law, M., Krauss, O., Hollinshead, M., and Smith, G.L. (2003). Vaccinia virus cores are transported on microtubules. J. Gen. Virol. *84*, 2443–2458.

Chou, W., Ngo, T., and Gershon, P.D. (2012). An overview of the vaccinia virus infectome: a survey of the proteins of the poxvirus-infected cell. J. Virol. *86*, 1487–1499.

Colamonici, O.R., Domanski, P., Sweitzer, S.M., Larner, A., and Buller, R.M. (1995). Vaccinia virus B18R gene encodes a type I interferon-binding protein that blocks interferon alpha transmembrane signaling. J. Biol. Chem. *270*, 15974–15978.

Croft, N.P., de Verteuil, D.A., Smith, S.A., Wong, Y.C., Schittenhelm, R.B., Tscharke, D.C., and Purcell, A.W. (2015). Simultaneous Quantification of Viral Antigen Expression Kinetics Using Data-Independent (DIA) Mass Spectrometry. Mol. Cell. Proteomics *14*, 1361–1372.

Daffis, S., Szretter, K.J., Schriewer, J., Li, J., Youn, S., Errett, J., Lin, T.Y., Schneller, S., Zust, R., Dong, H., et al. (2010). 2′-O methylation of the viral mRNA cap evades host restriction by IFIT family members. Nature *468*, 452–456.

de Magalhães, J.C., Andrade, A.A., Silva, P.N., Sousa, L.P., Ropert, C., Ferreira, P.C., Kroon, E.G., Gazzinelli, R.T., and Bonjardim, C.A. (2001). A mitogenic signal triggered at an early stage of vaccinia virus infection: implication of MEK/ERK and protein kinase A in virus multiplication. J. Biol. Chem. *276*, 38353–38360.

Elias, J.E., and Gygi, S.P. (2007). Target-decoy search strategy for increased confidence in large-scale protein identifications by mass spectrometry. Nat. Methods *4*, 207–214.

Elias, J.E., and Gygi, S.P. (2010). Target-decoy search strategy for mass spectrometry-based proteomics. Methods Mol. Biol. *604*, 55–71.

Everett, R.D., Bell, A.J., Lu, Y., and Orr, A. (2013). The replication defect of ICP0-null mutant herpes simplex virus 1 can be largely complemented by the combined activities of human cytomegalovirus proteins IE1 and pp71. J. Virol. *87*, 978–990.

Fenner, F., Henderson, D.A., Arita, I., Jezek, Z., and Ladnyi, I.D. (1988). Smallpox and Its Eradication (World Health Organization).

Goebel, S.J., Johnson, G.P., Perkus, M.E., Davis, S.W., Winslow, J.P., and Paoletti, E. (1990). The complete DNA sequence of vaccinia virus. Virology *179*, 247–266.

Grossegesse, M., Doellinger, J., Fritsch, A., Laue, M., Piesker, J., Schaade, L., and Nitsche, A. (2018). Global ubiquitination analysis reveals extensive modification and proteasomal degradation of cowpox virus proteins, but preservation of viral cores. Sci. Rep. *8*, 1807.

Guerin, J.L., Gelfi, J., Boullier, S., Delverdier, M., Bellanger, F.A., Bertagnoli, S., Drexler, I., Sutter, G., and Messud-Petit, F. (2002). Myxoma virus leukemia-associated protein is responsible for major histocompatibility complex class I and Fas-CD95 down-regulation and defines scrapins, a new group of surface cellular receptor abductor proteins. J. Virol. *76*, 2912–2923.

Haas, W., Faherty, B.K., Gerber, S.A., Elias, J.E., Beausoleil, S.A., Bakalarski, C.E., Li, X., Villén, J., and Gygi, S.P. (2006). Optimization and use of peptide mass measurement accuracy in shotgun proteomics. Mol. Cell. Proteomics *5*, 1326–1337.

Hollinshead, M., Johns, H.L., Sayers, C.L., Gonzalez-Lopez, C., Smith, G.L., and Elliott, G. (2012). Endocytic tubules regulated by Rab GTPases 5 and 11 are used for envelopment of herpes simplex virus. EMBO J. *31*, 4204–4220.

Hruby, D.E., and Ball, L.A. (1982). Mapping and identification of the vaccinia virus thymidine kinase gene. J. Virol. *43*, 403–409.

Hsu, J.L., van den Boomen, D.J., Tomasec, P., Weekes, M.P., Antrobus, R., Stanton, R.J., Ruckova, E., Sugrue, D., Wilkie, G.S., Davison, A.J., et al. (2015). Plasma membrane profiling defines an expanded class of cell surface proteins selectively targeted for degradation by HCMV US2 in cooperation with UL141. PLoS Pathog. *11*, e1004811.

Huang da, W., Sherman, B.T., and Lempicki, R.A. (2009). Systematic and integrative analysis of large gene lists using DAVID bioinformatics resources. Nat. Protoc. *4*, 44–57.

Hunter, S., Jones, P., Mitchell, A., Apweiler, R., Attwood, T.K., Bateman, A., Bernard, T., Binns, D., Bork, P., Burge, S., et al. (2012). InterPro in 2011: new developments in the family and domain prediction database. Nucleic Acids Res. *40*, D306–D312.

Huttlin, E.L., Jedrychowski, M.P., Elias, J.E., Goswami, T., Rad, R., Beausoleil, S.A., Villén, J., Haas, W., Sowa, M.E., and Gygi, S.P. (2010). A tissue-specific atlas of mouse protein phosphorylation and expression. Cell *143*, 1174–1189.

Jacobs, N., Bartlett, N.W., Clark, R.H., and Smith, G.L. (2008). Vaccinia virus lacking the Bcl-2-like protein N1 induces a stronger natural killer cell response to infection. J. Gen. Virol. *89*, 2877–2881.

Jones, E.V., and Moss, B. (1984). Mapping of the vaccinia virus DNA polymerase gene by marker rescue and cell-free translation of selected RNA. J. Virol. *49*, 72–77.

Käll, L., Canterbury, J.D., Weston, J., Noble, W.S., and MacCoss, M.J. (2007). Semi-supervised learning for peptide identification from shotgun proteomics datasets. Nat. Methods *4*, 923–925.

Kane, E.M., and Shuman, S. (1992). Temperature-sensitive mutations in the vaccinia virus H4 gene encoding a component of the virion RNA polymerase. J. Virol. *66*, 5752–5762.

Keck, J.G., Baldick, C.J., Jr., and Moss, B. (1990). Role of DNA replication in vaccinia virus gene expression: a naked template is required for transcription of three late trans-activator genes. Cell *61*, 801–809.

Kim, W., Bennett, E.J., Huttlin, E.L., Guo, A., Li, J., Possemato, A., Sowa, M.E., Rad, R., Rush, J., Comb, M.J., et al. (2011). Systematic and quantitative assessment of the ubiquitin-modified proteome. Mol. Cell *44*, 325–340.

Lebbink, R.J., van den Berg, M.C., de Ruiter, T., Raynal, N., van Roon, J.A., Lenting, P.J., Jin, B., and Meyaard, L. (2008). The soluble leukocyte-associated Ig-like receptor (LAIR)-2 antagonizes the collagen/LAIR-1 inhibitory immune interaction. J. Immunol. *180*, 1662–1669.

Li, C., Du, S., Tian, M., Wang, Y., Bai, J., Tan, P., Liu, W., Yin, R., Wang, M., Jiang, Y., et al. (2018). The Host Restriction Factor Interferon-Inducible Transmembrane Protein 3 Inhibits Vaccinia Virus Infection. Front. Immunol. *9*, 228.

Liu, B., Li, N.L., Wang, J., Shi, P.Y., Wang, T., Miller, M.A., and Li, K. (2014). Overlapping and distinct molecular determinants dictating the antiviral activities of TRIM56 against flaviviruses and coronavirus. J. Virol. *88*, 13821–13835.

Liu, B., Li, N.L., Shen, Y., Bao, X., Fabrizio, T., Elbahesh, H., Webby, R.J., and Li, K. (2016). The C-Terminal Tail of TRIM56 Dictates Antiviral Restriction of Influenza A and B Viruses by Impeding Viral RNA Synthesis. J. Virol. *90*, 4369–4382.

Liu, R., and Moss, B. (2018). Vaccinia Virus C9 Ankyrin Repeat/F-Box Protein Is a Newly Identified Antagonist of the Type I Interferon-Induced Antiviral State. J. Virol. *92*, e00053-18.

Liu, X., Wang, Q., Pan, Y., and Wang, C. (2015). Sensing and responding to cytosolic viruses invasions: An orchestra of kaleidoscopic ubiquitinations. Cytokine Growth Factor Rev. *26*, 379–387.

Lomonte, P., Thomas, J., Texier, P., Caron, C., Khochbin, S., and Epstein, A.L. (2004). Functional interaction between class II histone deacetylases and ICP0 of herpes simplex virus type 1. J. Virol. *78*, 6744–6757.

Makarov, A., and Denisov, E. (2009). Dynamics of ions of intact proteins in the Orbitrap mass analyzer. J. Am. Soc. Mass Spectrom. *20*, 1486–1495.

McAlister, G.C., Huttlin, E.L., Haas, W., Ting, L., Jedrychowski, M.P., Rogers, J.C., Kuhn, K., Pike, I., Grothe, R.A., Blethrow, J.D., and Gygi, S.P. (2012). Increasing the multiplexing capacity of TMTs using reporter ion isotopologues with isobaric masses. Anal. Chem. *84*, 7469–7478.

McAlister, G.C., Nusinow, D.P., Jedrychowski, M.P., Wühr, M., Huttlin, E.L., Erickson, B.K., Rad, R., Haas, W., and Gygi, S.P. (2014). MultiNotch MS3 enables accurate, sensitive, and multiplexed detection of differential expression across cancer cell line proteomes. Anal. Chem. *86*, 7150–7158.

McSharry, B.P., Jones, C.J., Skinner, J.W., Kipling, D., and Wilkinson, G.W. (2001). Human telomerase reverse transcriptase-immortalized MRC-5 and HCA2 human fibroblasts are fully permissive for human cytomegalovirus. J. Gen. Virol. *82*, 855–863.

Meade, N., Furey, C., Li, H., Verma, R., Chai, Q., Rollins, M.G., DiGiuseppe, S., Naghavi, M.H., and Walsh, D. (2018). Poxviruses Evade Cytosolic Sensing through Disruption of an mTORC1-mTORC2 Regulatory Circuit. Cell *174*, 1143–1157.e17.

Mercer, J., Snijder, B., Sacher, R., Burkard, C., Bleck, C.K., Stahlberg, H., Pelkmans, L., and Helenius, A. (2012). RNAi screening reveals proteasome- and Cullin3-dependent stages in vaccinia virus infection. Cell Rep. *2*, 1036–1047.

Moss, B. (1968). Inhibition of HeLa cell protein synthesis by the vaccinia virion. J. Virol. *2*, 1028–1037.

Moss, B. (2013). Poxviridae. In Fields Virology, D.M. Knipe and P.M. Howley, eds. (Lippincott Williams & Wilkins), pp. 2129–2159.

Najarro, P., Traktman, P., and Lewis, J.A. (2001). Vaccinia virus blocks gamma interferon signal transduction: viral VH1 phosphatase reverses Stat1 activation. J. Virol. *75*, 3185–3196.

Nightingale, K., Lin, K.M., Ravenhill, B.J., Davies, C., Nobre, L., Fielding, C.A., Ruckova, E., Fletcher-Etherington, A., Soday, L., Nichols, H., et al. (2018). High-Definition Analysis of Host Protein Stability during Human Cytomegalovirus Infection Reveals Antiviral Factors and Viral Evasion Mechanisms. Cell Host Microbe *24*, 447–460.e11.

Nusinzon, I., and Horvath, C.M. (2003). Interferon-stimulated transcription and innate antiviral immunity require deacetylase activity and histone deacetylase 1. Proc. Natl. Acad. Sci. USA *100*, 14742–14747.

Parkinson, J.E., and Smith, G.L. (1994). Vaccinia virus gene A36R encodes a M(r) 43-50 K protein on the surface of extracellular enveloped virus. Virology *204*, 376–390.

Parrish, S., and Moss, B. (2006). Characterization of a vaccinia virus mutant with a deletion of the D10R gene encoding a putative negative regulator of gene expression. J. Virol. *80*, 553–561.

Parrish, S., and Moss, B. (2007). Characterization of a second vaccinia virus mRNA-decapping enzyme conserved in poxviruses. J. Virol. *81*, 12973–12978.

Parrish, S., Resch, W., and Moss, B. (2007). Vaccinia virus D10 protein has mRNA decapping activity, providing a mechanism for control of host and viral gene expression. Proc. Natl. Acad. Sci. USA *104*, 2139–2144.

Pichlmair, A., Lassnig, C., Eberle, C.A., Górna, M.W., Baumann, C.L., Burkard, T.R., Bürckstümmer, T., Stefanovic, A., Krieger, S., Bennett, K.L., et al. (2011). IFIT1 is an antiviral protein that recognizes 5′-triphosphate RNA. Nat. Immunol. *12*, 624–630.

Portal, D., Rosendorff, A., and Kieff, E. (2006). Epstein-Barr nuclear antigen leader protein coactivates transcription through interaction with histone deacetylase 4. Proc. Natl. Acad. Sci. USA *103*, 19278–19283.

Rahm, N., and Telenti, A. (2012). The role of tripartite motif family members in mediating susceptibility to HIV-1 infection. Curr. Opin. HIV AIDS *7*, 180–186.

Ran, F.A., Hsu, P.D., Wright, J., Agarwala, V., Scott, D.A., and Zhang, F. (2013). Genome engineering using the CRISPR-Cas9 system. Nat. Protoc. *8*, 2281–2308.

Rice, A.P., and Roberts, B.E. (1983). Vaccinia virus induces cellular mRNA degradation. J. Virol. *47*, 529–539.

Rosel, J., and Moss, B. (1985). Transcriptional and translational mapping and nucleotide sequence analysis of a vaccinia virus gene encoding the precursor of the major core polypeptide 4b. J. Virol. *56*, 830–838.

Sanz, P., and Moss, B. (1999). Identification of a transcription factor, encoded by two vaccinia virus early genes, that regulates the intermediate stage of viral gene expression. Proc. Natl. Acad. Sci. USA *96*, 2692–2697.

Satheshkumar, P.S., Anton, L.C., Sanz, P., and Moss, B. (2009). Inhibition of the ubiquitin-proteasome system prevents vaccinia virus DNA replication and expression of intermediate and late genes. J. Virol. *83*, 2469–2479.

Schoggins, J.W., Wilson, S.J., Panis, M., Murphy, M.Y., Jones, C.T., Bieniasz, P., and Rice, C.M. (2011). A diverse range of gene products are effectors of the type I interferon antiviral response. Nature *472*, 481–485.

Schreiner, S., and Wodrich, H. (2013). Virion factors that target Daxx to overcome intrinsic immunity. J. Virol. *87*, 10412–10422.

Seo, G.J., Kim, C., Shin, W.J., Sklan, E.H., Eoh, H., and Jung, J.U. (2018). TRIM56-mediated monoubiquitination of cGAS for cytosolic DNA sensing. Nat. Commun. *9*, 613.

Silva, P.N., Soares, J.A., Brasil, B.S., Nogueira, S.V., Andrade, A.A., de Magalhães, J.C., Bonjardim, M.B., Ferreira, P.C., Kroon, E.G., Bruna-Romero, O., and Bonjardim, C.A. (2006). Differential role played by the MEK/ERK/EGR-1 pathway in orthopoxviruses vaccinia and cowpox biology. Biochem. J. *398*, 83–95.

Smith, G.L., Chan, Y.S., and Kerr, S.M. (1989a). Transcriptional mapping and nucleotide sequence of a vaccinia virus gene encoding a polypeptide with extensive homology to DNA ligases. Nucleic Acids Res. *17*, 9051–9062.

Smith, G.L., de Carlos, A., and Chan, Y.S. (1989b). Vaccinia virus encodes a thymidylate kinase gene: sequence and transcriptional mapping. Nucleic Acids Res. *17*, 7581–7590.

Smith, G.L., Talbot-Cooper, C., and Lu, Y. (2018). How Does Vaccinia Virus Interfere With Interferon? Adv. Virus Res. *100*, 355–378.

Smith, W., Tomasec, P., Aicheler, R., Loewendorf, A., Nemčovičová, I., Wang, E.C., Stanton, R.J., Macauley, M., Norris, P., Willen, L., et al. (2013). Human cytomegalovirus glycoprotein UL141 targets the TRAIL death receptors to thwart host innate antiviral defenses. Cell Host Microbe *13*, 324–335.

Stanton, R.J., McSharry, B.P., Rickards, C.R., Wang, E.C., Tomasec, P., and Wilkinson, G.W. (2007). Cytomegalovirus destruction of focal adhesions revealed in a high-throughput Western blot analysis of cellular protein expression. J. Virol. *81*, 7860–7872.

Strnadova, P., Ren, H., Valentine, R., Mazzon, M., Sweeney, T.R., Brierley, I., and Smith, G.L. (2015). Inhibition of Translation Initiation by Protein 169: A Vaccinia Virus Strategy to Suppress Innate and Adaptive Immunity and Alter Virus Virulence. PLoS Pathog. *11*, e1005151.

Stuart, J.H., Sumner, R.P., Lu, Y., Snowden, J.S., and Smith, G.L. (2016). Vaccinia Virus Protein C6 Inhibits Type I IFN Signalling in the Nucleus and Binds to the Transactivation Domain of STAT2. PLoS Pathog. *12*, e1005955.

Symons, J.A., Alcamí, A., and Smith, G.L. (1995). Vaccinia virus encodes a soluble type I interferon receptor of novel structure and broad species specificity. Cell *81*, 551–560.

Teale, A., Campbell, S., Van Buuren, N., Magee, W.C., Watmough, K., Couturier, B., Shipclark, R., and Barry, M. (2009). Orthopoxviruses require a functional ubiquitin-proteasome system for productive replication. J. Virol. *83*, 2099–2108.

Timms, R.T., Duncan, L.M., Tchasovnikarova, I.A., Antrobus, R., Smith, D.L., Dougan, G., Weekes, M.P., and Lehner, P.J. (2013). Haploid genetic screens identify an essential role for PLP2 in the downregulation of novel plasma membrane targets by viral E3 ubiquitin ligases. PLoS Pathog. *9*, e1003772.

Ting, L., Rad, R., Gygi, S.P., and Haas, W. (2011). MS3 eliminates ratio distortion in isobaric multiplexed quantitative proteomics. Nat. Methods *8*, 937–940.

Tscharke, D.C., Reading, P.C., and Smith, G.L. (2002). Dermal infection with vaccinia virus reveals roles for virus proteins not seen using other inoculation routes. J. Gen. Virol. *83*, 1977–1986.

Unterholzner, L., Sumner, R.P., Baran, M., Ren, H., Mansur, D.S., Bourke, N.M., Randow, F., Smith, G.L., and Bowie, A.G. (2011). Vaccinia virus protein C6 is a virulence factor that binds TBK-1 adaptor proteins and inhibits activation of IRF3 and IRF7. PLoS Pathog. *7*, e1002247.

Vivier, E., Tomasello, E., Baratin, M., Walzer, T., and Ugolini, S. (2008). Functions of natural killer cells. Nat. Immunol. *9*, 503–510.

Vladimer, G.I., Górna, M.W., and Superti-Furga, G. (2014). IFITs: Emerging Roles as Key Anti-Viral Proteins. Front. Immunol. *5*, 94.

Vos, J.C., and Stunnenberg, H.G. (1988). Derepression of a novel class of vaccinia virus genes upon DNA replication. EMBO J. *7*, 3487–3492.

Wali, A., and Strayer, D.S. (1999). Infection with vaccinia virus alters regulation of cell cycle progression. DNA Cell Biol. *18*, 837–843.

Weekes, M.P., Tomasec, P., Huttlin, E.L., Fielding, C.A., Nusinow, D., Stanton, R.J., Wang, E.C., Aicheler, R., Murrell, I., Wilkinson, G.W., et al. (2014). Quantitative temporal viromics: an approach to investigate host-pathogen interaction. Cell *157*, 1460–1472.

Wilcock, D., Duncan, S.A., Traktman, P., Zhang, W.H., and Smith, G.L. (1999). The vaccinia virus A4OR gene product is a nonstructural, type II membrane glycoprotein that is expressed at the cell surface. J. Gen. Virol. *80*, 2137–2148.

Wu, R., Dephoure, N., Haas, W., Huttlin, E.L., Zhai, B., Sowa, M.E., and Gygi, S.P. (2011). Correct interpretation of comprehensive phosphorylation dynamics requires normalization by protein expression changes. Mol. Cell Proteomics *10*, M111.009654.

Yang, Z., Bruno, D.P., Martens, C.A., Porcella, S.F., and Moss, B. (2010). Simultaneous high-resolution analysis of vaccinia virus and host cell transcriptomes by deep RNA sequencing. Proc. Natl. Acad. Sci. USA *107*, 11513–11518.

Yang, Z., Bruno, D.P., Martens, C.A., Porcella, S.F., and Moss, B. (2011a). Genome-wide analysis of the 5′ and 3′ ends of vaccinia virus early mRNAs delineates regulatory sequences of annotated and anomalous transcripts. J. Virol. *85*, 5897–5909.

Yang, Z., Reynolds, S.E., Martens, C.A., Bruno, D.P., Porcella, S.F., and Moss, B. (2011b). Expression profiling of the intermediate and late stages of poxvirus replication. J. Virol. *85*, 9899–9908.

Yang, Z., Cao, S., Martens, C.A., Porcella, S.F., Xie, Z., Ma, M., Shen, B., and Moss, B. (2015). Deciphering poxvirus gene expression by RNA sequencing and ribosome profiling. J. Virol. *89*, 6874–6886.

Yoo, N.K., Pyo, C.W., Kim, Y., Ahn, B.Y., and Choi, S.Y. (2008). Vaccinia virus-mediated cell cycle alteration involves inactivation of tumour suppressors associated with Brf1 and TBP. Cell. Microbiol. *10*, 583–592.

STAR★METHODS

KEY RESOURCES TABLE

REAGENT or RESOURCE	SOURCE	IDENTIFIER
Antibodies		
Rabbit polyclonal anti-IFIT1	Thermo Fisher Scientific	Cat#PA3-848; RRID:AB_1958733
Mouse monoclonal anti-IFITM1/2/3	Santa Cruz Biotechnology	Cat#sc-374026; RRID:AB_10916884
Mouse monoclonal anti-COL6A2	Santa Cruz Biotechnology	Cat#sc-374566; RRID:AB_10991101
Mouse monoclonal anti-COL1A2	Santa Cruz Biotechnology	Cat#sc-376350; RRID:AB_10989920
Mouse monoclonal anti-TRIM5	Santa Cruz Biotechnology	Cat#sc-373864; RRID:AB_10918111
Mouse monoclonal anti-HDAC5	Santa Cruz Biotechnology	Cat#sc-133225; RRID:AB_2116791
Rat monoclonal anti-α-tubulin (clone YL1/2)	Serotec	Cat#MCA77G; RRID:AB_325003
Rabbit polyclonal anti-C6	Unterholzner et al., 2011	N/A
Mouse monoclonal anti-D8 (clone AB1.1)	Parkinson and Smith, 1994	N/A
IRDye 680RD-conjugated goat anti-rabbit IgG	LI-COR	Cat#926-68071; RRID:AB_10956166
IRDye 680LT-conjugated goat anti-mouse IgG	LI-COR	Cat#926-68020; RRID:AB_10706161
IRDye 800CW-conjugated goat anti-rabbit IgG	LI-COR	Cat#926-32211; RRID:AB_621843
IRDye 800CW-conjugated goat anti-mouse IgG	LI-COR	Cat#926-32210; RRID:AB_621842
IRDye 680LT-conjugated goat anti-rat IgG	LI-COR	Cat#926-68029; RRID:AB_10715073
PE goat anti-mouse IgG (clone Poly4053)	BioLegend	Cat#405307; RRID:AB_315010
Mouse monoclonal anti-HDAC1	Santa Cruz Biotechnology	Cat# sc-81598; RRID:AB_ 2118083
Mouse monoclonal anti-FLAG	Sigma-Aldrich	Cat# F3165; RRID:AB_259529
Mouse monoclonal anti-Myc	Cell Signaling Technology	Cat#2276; RRID:AB_331783
Bacterial and Virus Strains		
vC6WR (Western Reserve)	Laboratory of Geoffrey Smith; described in Unterholzner et al., 2011	N/A
vΔC6 (derived from Western Reserve)	Laboratory of Geoffrey Smith; described in Unterholzner et al., 2011	N/A
A5GFP VACV (derived from Western Reserve)	Laboratory of Geoffrey Smith; described in Carter et al., 2003	N/A
VP26GFP HSV-1 (derived from s17 strain)	Laboratory of Prashant Desai; described in Hollinshead et al., 2012	N/A
E. coli (Stbl3 Chemically Competent Cells)	Invitrogen	Cat#C737303
E. coli (Subcloning Efficiency DH5α Competent Cells)	Invitrogen	Cat#18265-017
Chemicals, Peptides, and Recombinant Proteins		
Tandem mass tag (TMT) 10-plex isobaric reagents	Thermo Fisher Scientific	Cat#90110
HPLC water	VWR	Cat#23595.328
LC-MS grade Acetonitrile	Merck	Cat#1.00029.2500
Formic acid	Thermo Fisher Scientific	Cat#85178
MG132	Abcam	Cat#ab141003
DMEM	GIBCO	Cat#41966-029
MEM	GIBCO	Cat#31095-029
FBS	PAN Biotech	Cat#P30-19375
Penicillin/streptomycin	GIBCO	Cat#15140-122
Cytosine arabinoside (AraC)	Sigma-Aldrich	Cat#C6645
Polybrene	Sigma-Aldrich	Cat#H9268
G418	BioVision	Cat#1557
Puromycin	InvivoGen	Cat#ant-pr-1

(*Continued on next page*)

Continued

REAGENT or RESOURCE	SOURCE	IDENTIFIER
Trypsin-EDTA	GIBCO	Cat#25300-054
Bovine serum albumin	Sigma-Aldrich	Cat#A7906
Saponin	Sigma-Aldrich	Cat#S4521
HEPES (1M, pH 7.0-7.6)	Sigma-Aldrich	Cat#H0887
Guanidine hydrochloride (8M)	Thermo Fisher Scientific	Cat#24115
DL-Dithiothreitol	Sigma	Cat# 43815-1G
Iodoacetamide	Sigma	Cat# I1149-5G
Lysyl Endopeptidase	Wako	Cat# 125-02543
Trypsin Protease	Pierce	Cat# 90058
Hydroxylamine	Sigma	Cat# 438227
Acetonitrile, Extra Dry	Acros Organics	Cat# AC364311000
cOmplete, EDTA-free Protease Inhibitor Cocktail	Roche	Cat#11836153001
PhosSTOP Phosphatase Inhibitor Cocktail	Roche	Cat#04906837001
Paraformaldehyde, 16% solution, EM grade	Electron Microscopy Sciences	Cat#15710-S
Carbenicillin	Sigma-Aldrich	Cat#C9231
Critical Commercial Assays		
Micro BCA Protein Assay Kit	Thermo Fisher Scientific	Cat#23235
TOPO TA Cloning Kit for Sequencing	Invitrogen	Cat#K4575J10
Pierce BCA Protein Assay Kit	Thermo Fisher Scientific	Cat#23227
Platinum *Taq* DNA Polymerase High Fidelity	Invitrogen	Cat#11304011
QIAamp DNA Mini Kit	QIAGEN	Cat#51306
Deposited Data		
Unprocessed peptide files	This paper	https://doi.org/10.17632/wxk9gnw22r.1
Raw Mass Spectrometry Data Files	This paper	ProteomeXchange Consortium via the PRIDE partner repository, with the dataset identifier PXD012785.
Experimental Models: Cell Lines		
Human fetal foreskin fibroblasts (HFFFs) immortalized with human telomerase (HFFF-TERTs)	Stanton et al., 2007	N/A
BS-C-1 (African green monkey cell line)	ATCC	ATCC: CCL-26
RK_{13} cells (rabbit kidney cell line)	ATCC	ATCC: CCL-37
HEK293T (human embryo kidney epithelial cell line)	ATCC	ATCC: CRL-11268
HeLa (human cervical adenocarcinoma epithelial cell line)	ATCC	ATCC: CCL-2
U-2 OS (human osteosarcoma epithelial cell line)	ATCC	ATCC: HTB-96
$HDAC5^{-/-}$ HeLa cell line (clones KO1 and KO2)	This paper	N/A
$HDAC5^{-/-}$ HEK293T cell line (clones KO3 and KO4)	This paper	N/A
Oligonucleotides		
Forward primer for exon 3 of human HDAC5: AGTGGCCTGAGGGAACCTGTGCTGT	This paper	N/A
Reverse primer for exon 3 of human HDAC5: AGGCAGGGACATCAAGGCACTTAC	This paper	N/A
Forward primer for exon 4 of human HDAC5: AAAATGTTGCATCCATGGAGCAG	This paper	N/A
Reverse primer for exon 4 of human HDAC5: ATGGGAACGGAGGCACAAGTGA	This paper	N/A
Complete oligonucleotides including those for CRISPR/cas9 gene disruption.	Table S7	N/A

(*Continued on next page*)

Continued		
REAGENT or RESOURCE	SOURCE	IDENTIFIER
Recombinant DNA		
pSpCas9(BB)-2A-Puro (px459)	Ran et al., 2013	Addgene plasmid #62988
pLKOneo.EGFPnlsTetR	Laboratory of Roger Everett; Everett et al., 2013	N/A
pLKO.DCMV.TetO.mcs	Laboratory of Roger Everett; Everett et al., 2013	N/A
pLKO.DCMV.TetO.HDAC5-FLAG	This paper	N/A
pCMV.dR8.91	Laboratory of Heike Laman	N/A
pMD-G	Laboratory of Heike Laman	N/A
Software and Algorithms		
"MassPike," a Sequest-based software pipeline for quantitative proteomics.	Professor Steven Gygi's lab, Harvard Medical School, Boston, USA.	N/A
XLStat	Addinsoft	https://www.xlstat.com/en/
DAVID software	Huang da et al., 2009	https://david.ncifcrf.gov/
Cluster 3.0	Stanford University of Tokyo	http://bonsai.hgc.jp/~mdehoon/software/cluster/software.htm
Java Treeview	SourceForge.net	http://jtreeview.sourceforge.net/
Clustal Omega	EMBL-EBI	https://www.ebi.ac.uk/Tools/msa/clustalo/
FlowJo CE (version 7.5.109.8)	FlowJo	https://www.flowjo.com/solutions/flowjo
FlowJo (version 10.1r5)	FlowJo	https://www.flowjo.com/solutions/flowjo
Perseus (version 1.5.1.6)	Max Planck Institute of Biochemistry	http://www.coxdocs.org/doku.php?id=perseus:start
GraphPad Prism 5 for Windows (version 5.04)	GraphPad Software	https://www.graphpad.com/
Odyssey Infrared Imaging System (version 3.0.29)	LI-COR Biosciences	http://www.licor.com/bio/blog/category/imaging-systems/odyssey-imaging-systems/
AxioVision (version 4.8)	ZEISS	https://www.zeiss.com/microscopy/us/downloads/axiovision-downloads.html
Other		
Orbitrap Fusion Lumos Mass Spectrometer	Thermo Fisher Scientific	Cat#IQLAAEGAAP FADBMBHQ
Trans-Blot Turbo Transfer System	Bio-Rad	Cat#1704150
FACScan flow cytometry analyzer, upgraded to DxP8 by Cytek	FACScan/Cytek	N/A
Odyssey Infrared Imaging System	LI-COR Biosciences	Cat#9120
ZEISS Axio Vert.A1 fluorescence microscope	ZEISS	Cat# 491237-0014-000

CONTACT FOR REAGENT AND RESOURCE SHARING

Further information and requests for resources and reagents should be directed to and will be fulfilled by the Lead Contact, Michael Weekes (mpw1001@cam.ac.uk).

EXPERIMENTAL MODEL AND SUBJECT DETAILS

Cells and Cell Culture

Primary human fetal foreskin fibroblast cells immortalized with human telomerase (HFFF-TERTs, male), BS-C-1 (African green monkey cell line, ATCC CCL-26), HeLa (human cervical ATCC CCL-2,+ female) parental and HDAC5$^{-/-}$ derivative cell lines, HEK293T (human embryo kidney epithelial cell line, ATCC CRL-11268, female) parental and HDAC5$^{-/-}$ derivative cell lines and human bone osteosarcoma epithelial (U2OS, ATCC HTB-96, female) cells were grown in Dulbecco's modified Eagle's medium (DMEM) supplemented with fetal bovine serum (FBS: 10% v/v), and penicillin/streptomycin at 37°C in 5% CO_2. Transduced U2OS.TetR.HDAC5-FLAG, HEK.TetR.TAPcoC6 and HEK.TetR.MyccoC6 cell lines were additionally supplemented with 500μg/ml G418 and 1 μg/ml puromycin. RK_{13} cells (rabbit kidney cell line, ATCC CCL-37) were maintained in minimal essential medium (MEM) supplemented with 10 % FBS and penicillin/streptomycin.

All cell lines apart from HFFF-TERTs were obtained from and authenticated by ATCC. HFFF-TERTs have been tested at regular intervals since isolation to confirm both that the HLA and MICA genotypes, and the morphology and antibiotic resistances are consistent with the original cells described in (McSharry et al., 2001). In addition, HFFF-TERTs are routinely infected with the HCMV Merlin strain, which is only permissive in human fibroblasts (dermal or foreskin), further limiting the chances that the cells have been contaminated with another cell type. HDAC5-deficient HeLa and HEK cell lines were derived from the respective parental cell lines obtained from ATCC. All cell lines used regularly tested negative for mycoplasma.

Viruses

Wild-type VACV strain Western Reserve (WR) and a derivative strain lacking the *C6L* gene, or with the capsid protein A5 fused with GFP (A5GFP VACV) were described (Carter et al., 2003; Unterholzner et al., 2011). HSV-1 strain s17 with GFP fused to virus protein 26 (VP26GFP) was provided by Prashant Desai (Hollinshead et al., 2012). VACVs were propagated in RK_{13} cells, purified by ultracentrifugation through a 36% (w/v) sucrose cushion and suspended in 10 mM Tris-HCl pH 9.0. VACV infectivity was determined by plaque assay on BS-C-1 cells. VP26GFP HSV-1 was propagated in U2OS cells, and the infectivity was determined by plaque assay on U2OS cells.

Plasmids

The CRISPR/Cas9 plasmid px459 was from Addgene, #62988. Lentivirus vector plasmids pLKOneo.EGFPnlsTetR and pLKO.DCMV.TetO.mcs (Everett et al., 2013) were from Prof. Roger Everett (MRC, Centre for Virus Research, University of Glasgow). Plasmid pLKO.DCMV.TetO.HDAC5-FLAG was constructed by insertion of HDAC5-FLAG sequence with a 5′ HindIII site and a 3′MluI into the multiple cloning site of pLKO.DCMV.TetO.mcs. Plasmids pLKO.DCMV.TetO.TAPcoC6 and pLKO.DCMV.TetO.MyccoC6 were constructed by insertion of TAPcoC6 (carboxyl terminus TAP tag) or MyccoC6 (amino-terminus Myc tag) sequence with a 5′ Sal1 site and a 3′ EcoR1 into the multiple cloning site of pLKO.DCMV.TetO.mcs. Plasmids pCMV.dR8.91 (expressing lentivirus helper functions) and pMD-G (vesicular stomatitis virus envelope protein G) were from Heike Laman (Department of Pathology, University of Cambridge) and were used together with the above lentivirus vectors, to produce lentivirus stocks as described (Everett et al., 2013).

METHOD DETAILS

Virus Infections and Inhibitors

For proteomic experiments, 1×10^6 HFFF-TERTs were seeded in a 25-cm^2 flask. Cells were infected at MOI 5. All time course experiments (WCL1-3) were performed in biological triplicate. Where indicated cells were incubated with cytosine arabinoside (AraC) at 40 μg/ml from the time of infection. For the experiment comparing wt infection with the derivative lacking the *C6L*, and wt infection in the presence or absence of MG132 (‘wt_C6L_MG’), where appropriate, after 2 h, inocula were removed and replaced with fresh medium with or without 10 μM MG132. This experiment was conducted in biological triplicate for wt(-MG132), wt(+MG132) and Δ*C6L*, and in single replicates for mock infection ± MG132 to fit the 11-plex limit of TMT experiments (Table S7B). For immunoblots, 1×10^6 HFFF-TERTs were plated in 6-well plates. Cells were infected or mock infected at MOI 5. After 2 h, inocula were removed and replaced with fresh medium with or without 10 μM MG132.

For virus replication assays, 2×10^6 parental or HDAC5$^{-/-}$ HEK293T cells were seeded in 6-well plates. Cells were infected at MOI 0.001 with A5GFP VACV. After 2 d, both the supernatant and infected cells were collected for titration on BS-C-1 cells. Thus, in Figure 7G, virus yield is shown without distinction between intracellular and extracellular virions. VP26GFP HSV-1 infections were as above, except supernatants were collected at 3 d p.i. for titration on U2OS. Similar virus infection assays were performed on parental and HDAC5$^{-/-}$ HeLa cells.

To measure GFP positive foci, infected monolayers of parental or HDAC5$^{-/-}$ HeLa cells were infected for 2 d with VACV or 3 d with HSV-1 and then imaged at 50X magnification using a ZEISS Axio Vert.A1 fluorescent microscope and the AxioVision 4.8 software.

Lentiviral Transduction

HEK293T cells (3×10^6) were seeded in 10-cm dishes and on the following day were transfected with 3 μg of pLKOneo.EGFPnlsTetR together with 3 μg of each pMD-G and pCMV.dR8.91. After 3 h, the cell culture medium was replaced with DMEM supplemented with 30% FBS and penicillin-streptomycin. After overnight incubation, the cell culture supernatant was collected and replaced with 5 mL of 30% FBS/DMEM. The collected supernatant was passed through a 0.45 μm filter and supplemented with 2 μg/ml polybrene (Sigma-Aldrich, H9268). Lentivirus stock was used to transduce U2OS cells over two successive days. Transduced cells were cultured for 2 days, then the cells were selected with 500 μg/ml G418 (BioVision, 1557), to obtain a cell line stably expressing TetR repressor. The same method was used to introduce HDAC5-FLAG into different cell lines (U2OS-TetR and HDAC5$^{-/-}$) with the plasmid pLKO.TetO.HDAC5-FLAG. The HDAC5-FLAG transduced cells were selected with 1 μg/ml puromycin (InvivoGen, 58-58-2).

CRISPR/Cas9-Mediated Gene Knockout

px459 plasmids expressing gRNAs targeting HDAC5 exon 3 and exon 4 were prepared as described (Ran et al., 2013). HeLa or HEK293T cells were seeded in 10-cm dishes and the next day were transfected with 3 μg of either px459.HDAC5E4 or

px459.HDAC5E3, expressing gRNAs to target *HDAC5* exons 4 and 3, respectively (Table S7A). The following day, transfected cells were selected in medium with 1 μg/ml puromycin. After 2 days, selection medium was replaced with normal DMEM with 10% FBS. After 5-7 days, the cell population had reached 50%–80% confluence. Cells were detached, serially diluted and seeded into 96-well plates. Single-cell clones were selected for analysis. To confirm gene knockout, lysates from each clone were analyzed by immunoblotting and DNA sequencing of the genomic region targeted by each gRNA. For sequencing, primers were designed to be specific to introns flanking *HDAC5* exon 3 or exon 4 (Table S7A). To identify DNA sequences from each allele, TOPO cloning (Invitrogen, K4575J10) was performed with HDAC5 PCR fragments and 20 colonies from each knockout clone were sequenced. Two HDAC5$^{-/-}$ clones were generated from HeLa parental cells with gRNAs targeting either *HDAC5* exon 4 (H5KO1) or *HDAC5* exon 3 (H5KO2), and two HDAC5$^{-/-}$ clones were generated from HEK293T cells with gRNA targeting *HDAC5* exon4 (H5KO3 and H5KO4). The DNA sequences of the gRNA-targeted regions from the HDAC5$^{-/-}$ clones used are shown in Table S7A and confirmed that each allele of each clone contained frameshifting mutations and that wild-type sequences were not detected.

Immunoblotting

Cells were washed with PBS, and scraped in 400 μL cell lysis buffer (50 mM Tris-HCl pH 8.0, 150 mM NaCl, 1 mM EDTA, 10% glycerol, 0.5% Triton X-100, 0.1% NP-40), supplemented with protease and phosphatase inhibitors cocktails (Roche). Cell lysates were transferred to 1.5 mL eppendorf tubes and clarified by centrifugation at 17,000 *g* at 4°C for 15 min. Protein concentration was measured by BCA assay (Pierce) and protein extracts were reduced with 100 mM DTT in SDS-gel loading buffer for 5 min at 100°C. Equal protein amounts were separated by SDS-PAGE in 8% or 12% polyacrylamide gels, then transferred to nitrocellulose membranes using the Trans-Blot Turbo transfer system (Bio-Rad). The membranes were blocked in 5% non-fat milk in TBS containing 0.1% Tween-20 for 30 min at room temperature. The following primary antibodies were used at the indicated dilution, in blocking solution: rabbit anti-IFIT1 (1:1000, cat. no. PA3-848, Thermo Fisher Scientific), mouse anti-IFITM1/2/3 (1:500, cat. no. sc-374026, Santa Cruz Biotechnology), mouse anti-COL6A2 (1:500, cat. no. sc-374566, Santa Cruz Biotechnology), mouse anti-COL1A2 (1:500, cat. no. sc-376350, Santa Cruz Biotechnology), mouse anti-TRIM5 (1:500, cat. no. SC-373864, Santa Cruz Biotechnology), mouse anti-HDAC5 (1:500, cat. no. sc-133225, Santa Cruz Biotechnology), mouse anti-HDAC1 (1:500, cat. no. sc-81598, Santa Cruz Biotechnology), mouse anti-FLAG, (1:1000, F3165, Sigma-Aldrich), mouse anti-Myc (1:1000, cat. no. 2276, Cell Signaling Technology), rat anti-α-tubulin (1:10,000, clone YL1/2, cat. no. MCA77G, Serotec), rabbit anti-C6 (1:1000, described in (Unterholzner et al., 2011), mouse anti-D8 (1:1000, described in (Parkinson and Smith, 1994)). The secondary antibodies were from LI-COR Biosciences and were used at 1:10,000 dilution, in blocking solution: IRDye 680RD-conjugated goat anti-rabbit IgG (cat. no. 926-68071), IRDye 680LT-conjugated goat anti-mouse IgG (926-68020), IRDye 800CW-conjugated goat anti-rabbit IgG (926-32211), IRDye 800CW-conjugated goat anti-mouse IgG (926-32210), IRDye 680LT-conjugated goat anti-rat IgG (926-68029). The reactive bands were detected using an Odyssey infrared imager (LI-COR Biosciences).

Flow Cytometry

VACV-infected HFFF-TERTs were detached with trypsin-EDTA (GIBCO) 12 h post-infection, washed in PBS and fixed with 4% paraformaldehyde in PBS for 10 min at room temperature with intermittent agitation by vortexing. Fixed cells were collected by centrifugation and suspended in PBS containing 0.1% bovine serum albumin (Sigma). For intracellular staining of VACV protein D8, cells were permeabilised with 0.1% saponin (Sigma) in PBS and stained with the mouse monoclonal antibody AB1.1 specific for the VACV protein D8 (Parkinson and Smith, 1994) or isotype control, followed by PE goat anti-mouse IgG (Poly4053, BioLegend). Stained cells were fixed again with 1% paraformaldehyde in PBS. Data were acquired with a FACScan/Cytek DxP8-upgraded flow cytometry analyzer and analyzed with FlowJo software.

Whole Cell Lysate Protein Digestion

Cells were washed twice with PBS and 250 μL lysis buffer was added (6 M guanidine / 50mM HEPES pH 8.5). Cell lifters (Corning) were used to scrape cells in lysis buffer, which was removed to an eppendorf tube, vortexed extensively and then sonicated. Cell debris was removed by centrifuging at 21,000 *g* for 10 min, twice. Half of each sample was kept for subsequent analysis by immunoblot where required. For the other half, dithiothreitol (DTT) was added to a final concentration of 5 mM and samples were incubated for 20 min. Cysteines were alkylated with 14 mM iodoacetamide and incubated for 20 min at room temperature in the dark. Excess iodoacetamide was quenched with DTT for 15 min. Samples were diluted with 200 mM HEPES pH 8.5 to 1.5 M guanidine followed by digestion at room temperature for 3 h with LysC protease at a 1:100 protease-to-protein ratio. Samples were further diluted with 200 mM HEPES pH 8.5 to 0.5 M guanidine. Trypsin was then added at a 1:100 protease-to-protein ratio followed by overnight incubation at 37°C. The reaction was quenched with 5% formic acid and then centrifuged at 21,000 *g* for 10 min to remove undigested protein. Peptides were subjected to C18 solid-phase extraction (SPE, Sep-Pak, Waters) and vacuum-centrifuged to near-dryness.

Peptide Labeling with Tandem Mass Tags

In preparation for TMT labeling, desalted peptides were dissolved in 200 mM HEPES pH 8.5. Peptide concentration was measured by microBCA (Pierce), and 25 μg of peptide was labeled with TMT reagent. TMT reagents (0.8 mg) were dissolved in 43 μL anhydrous acetonitrile and 3 μL was added to peptide at a final acetonitrile concentration of 30% (v/v). Sample labeling was as indicated in Table S7B. Following incubation at room temperature for 1 h, the reaction was quenched with hydroxylamine to a final concentration of

0.5% (v/v). TMT-labeled samples were combined at a 1:1:1:1:1:1:1:1:1:1:1 ratio. The sample was vacuum-centrifuged to near dryness and subjected to C18 SPE (Sep-Pak, Waters). An unfractionated single shot was analyzed initially to ensure similar peptide loading across each TMT channel, to avoid the need for excessive electronic normalization. As all normalization factors were > 0.5 and < 2, data for the WCL2, WCL3 and wt_C6L_MG singleshot experiments were analyzed with data for the corresponding fractions to increase the overall number of peptides quantified. Normalization is discussed in the Data Analysis section below, and high pH reversed-phase (HpRP) fractionation is discussed below.

Offline HpRP Fractionation

TMT-labeled tryptic peptides were subjected to HpRP fractionation using an Ultimate 3000 RSLC UHPLC system (Thermo Fisher Scientific) equipped with a 2.1 mm internal diameter (ID) x 25 cm long, 1.7 μm particle Kinetix Evo C18 column (Phenomenex). Mobile phase consisted of A: 3% acetonitrile (MeCN), B: MeCN and C: 200 mM ammonium formate pH 10. Isocratic conditions were 90% A / 10% C, and C was maintained at 10% throughout the gradient elution. Separations were conducted at 45°C. Samples were loaded at 200 μl/min for 5 min. The flow rate was then increased to 400 μl/min over 5 min, after which the gradient elution proceed as follows: 0%–19% B over 10 min, 19%–34% B over 14.25 min, 34%–50% B over 8.75 min, followed by a 10 min wash at 90% B. UV absorbance was monitored at 280 nm and 15 s fractions were collected into 96-well microplates using the integrated fraction collector. Fractions were recombined orthogonally in a checkerboard fashion, combining alternate wells from each column of the plate into a single fraction, and commencing combination of adjacent fractions in alternating rows. Wells prior to the start or after the stop of elution of peptide-rich fractions, as identified from the UV trace, were excluded. This yielded two sets of 12 combined fractions, A and B, which were dried in a vacuum centrifuge and resuspended in 10 μl MS solvent (4% MeCN / 5% formic acid) prior to LC-MS3. For all experiments, 12 set 'A' fractions were used.

LC-MS3

Mass spectrometry data was acquired using an Orbitrap Lumos (Thermo Fisher Scientific, San Jose, CA). An Ultimate 3000 RSLC nano UHPLC equipped with a 300 μm ID x 5 mm Acclaim PepMap μ-Precolumn (Thermo Fisher Scientific) and a 75 μm ID x 50 cm 2.1 μm particle Acclaim PepMap RSLC analytical column was used. Loading solvent was 0.1% FA, analytical solvent A: 0.1% FA and B: 80% MeCN + 0.1% FA. All separations were carried out at 55°C. Samples were loaded at 5 μL/min for 5 min in loading solvent before beginning the analytical gradient. The following gradient was used: 3%–7% B over 3 min, 7%–37% B over 173 min, followed by a 4-min wash at 95% B and equilibration at 3% B for 15 min. Each analysis used a MultiNotch MS3-based TMT method (McAlister et al., 2012, 2014). The following settings were used: MS1: 380-1500 Th, 120,000 Resolution, 2×10^5 automatic gain control (AGC) target, 50 ms maximum injection time. MS2: Quadrupole isolation at an isolation width of m/z 0.7, CID fragmentation (normalized collision energy (NCE) 35) with ion trap scanning in turbo mode from m/z 120, $1.5\text{x}10^4$ AGC target, 120 ms maximum injection time. MS3: In Synchronous Precursor Selection mode the top 6 MS2 ions were selected for HCD fragmentation (NCE 65) and scanned in the Orbitrap at 60,000 resolution with an AGC target of 1×10^5 and a maximum accumulation time of 150 ms. Ions were not accumulated for all parallelisable time. The entire MS/MS/MS cycle had a target time of 3 s. Dynamic exclusion was set to ± 10 ppm for 70 s. MS2 fragmentation was trigged on precursors 5×10^3 counts and above.

QUANTIFICATION AND STATISTICAL ANALYSIS

Data Analysis

In the following description, we list the first report in the literature for each relevant algorithm. Mass spectra were processed using a Sequest-based software pipeline for quantitative proteomics, "MassPike," through a collaborative arrangement with Professor Steve Gygi's laboratory at Harvard Medical School. MS spectra were converted to mzXML using an extractor built upon Thermo Fisher's RAW File Reader library (version 4.0.26). In this extractor, the standard mzxml format has been augmented with additional custom fields that are specific to ion trap and Orbitrap mass spectrometry and essential for TMT quantitation. These additional fields include ion injection times for each scan, Fourier Transform-derived baseline and noise values calculated for every Orbitrap scan, isolation widths for each scan type, scan event numbers, and elapsed scan times. This software is a component of the MassPike software platform and is licensed by Harvard Medical School.

A combined database was constructed from (a) the human UniProt database (26th January, 2017), (b) the VACV strain WR UniProt database (23rd February 2017), (c) common contaminants such as porcine trypsin and endoproteinase LysC. The combined database was concatenated with a reverse database composed of all protein sequences in reversed order. Searches were performed using a 20 ppm precursor ion tolerance (Haas et al., 2006). Product ion tolerance was set to 0.03 Th. TMT tags on lysine residues and peptide N termini (229.162932 Da) and carbamidomethylation of cysteine residues (57.02146 Da) were set as static modifications, while oxidation of methionine residues (15.99492 Da) was set as a variable modification.

To control the fraction of erroneous protein identifications, a target-decoy strategy was employed (Elias and Gygi, 2007, 2010). Peptide spectral matches (PSMs) were filtered to an initial peptide-level false discovery rate (FDR) of 1% with subsequent filtering to attain a final protein-level FDR of 1% (Kim et al., 2011; Wu et al., 2011). PSM filtering was performed using a linear discriminant analysis, as described (Huttlin et al., 2010). This distinguishes correct from incorrect peptide IDs in a manner analogous to the widely used Percolator algorithm (Käll et al., 2007), though employing a distinct machine learning algorithm. The following parameters were

considered: XCorr, ΔCn, missed cleavages, peptide length, charge state, and precursor mass accuracy. Protein assembly was guided by principles of parsimony to produce the smallest set of proteins necessary to account for all observed peptides (Huttlin et al., 2010).

Proteins were quantified by summing TMT reporter ion counts across all matching peptide-spectral matches using "MassPike," as described (McAlister et al., 2012, 2014). A minimum one unique or shared peptide per protein was used for quantitation. Briefly, a 0.003 Th window around the theoretical m/z of each reporter ion (126, 127n, 127c, 128n, 128c, 129n, 129c, 130n, 130c, 131n, 131c) was scanned for ions, and the maximum intensity nearest to the theoretical m/z was used. The primary determinant of quantitation quality is the number of TMT reporter ions detected in each MS3 spectrum, which is directly proportional to the signal-to-noise (S:N) ratio observed for each ion (Makarov and Denisov, 2009). Conservatively, every individual peptide used for quantitation was required to contribute sufficient TMT reporter ions (minimum of ~1250 per spectrum) so that each on its own could be expected to provide a representative picture of relative protein abundance (McAlister et al., 2012). Additionally, an isolation specificity filter was employed to minimize peptide co-isolation (Ting et al., 2011). Peptide-spectral matches with poor quality MS3 spectra (more than 9 TMT channels missing and/or a combined S:N ratio of less than 250 across all TMT reporter ions) or no MS3 spectra at all were excluded from quantitation. Peptides meeting the stated criteria for reliable quantitation were then summed by parent protein, in effect weighting the contributions of individual peptides to the total protein signal based on their individual TMT reporter ion yields. Protein quantitation values were exported for further analysis in Excel.

For protein quantitation, reverse and contaminant proteins were removed, then each reporter ion channel was summed across all quantified proteins and normalized assuming equal protein loading across all channels. For further analysis and display in figures, fractional TMT signals were used (i.e., reporting the fraction of maximal signal observed for each protein in each TMT channel, rather than the absolute normalized signal intensity). This effectively corrected for differences in the numbers of peptides observed per protein. For all TMT experiments, normalized S:N values are presented in Table S1 ('Data' worksheet).

Hierarchical centroid clustering based on uncentered Pearson correlation was performed using Cluster 3.0 (Stanford University). XLStat (Addinsoft) was used to perform k-means clustering. Each cluster was subjected to hierarchical clustering using Cluster 3.0. Clusters were visualized using Java Treeview (http://jtreeview.sourceforge.net).

Statistical Analysis

The exact value of n within figures is indicated in the respective figure legends, and refers to the number of biological replicates. Blinding or sample-size estimation was not appropriate for this study. There were no inclusion criteria and no data was excluded.

Figures 1, 2, 3, 4, 5, 6D, S1, S2, S3, and S4*.* Time course experiments were conducted in biological triplicate (WCL1-3). For experiment wt_C6L_MG, infections with VACV (+/− MG132) and VACV lacking *C6L* were performed in biological triplicate. In this experiment, to fit the constraints of 11-plex TMT, mock samples ± MG132 were examined in single replicates (Table S7B).

For proteins quantified in all three replicates, a two-tailed Student's t test was used to estimate p values that each average fold change was significantly different to 1. Values were calculated in Excel and corrected for multiple hypothesis testing using the method of Benjamini-Hochberg using an Excel macro written by one of the authors. A corrected p value < 0.05 was considered statistically significant.

Figures 4, *S4.* XLStat (Addinsoft) was used to calculate the summed distance of each protein from its cluster centroid then perform k-means clustering. Each cluster was subjected to hierarchical clustering using Cluster 3.0 (Stanford University) (Figure 4A).

Figures 6E and 6F. Immunoblots were performed in biological triplicate. Two-tailed t tests were used to estimate p values that each mean quantified relative band intensity in the ΔC6 or wt VACV+MG132 was different from control. A p value < 0.05 was considered statistically significant.

Figure 7. p values were calculated using two-tailed t tests. A p value < 0.05 was considered statistically significant.

Pathway Analysis

The Database for Annotation, Visualization and Integrated Discovery (DAVID) version 6.8 was used to determine pathway enrichment (Huang da et al., 2009). Proteins regulated as indicated in the text were searched against a background of all human proteins quantified, using default settings.

DATA AND SOFTWARE AVAILABILITY

Unprocessed peptide data files for Figures 1, 2, 3, 4, 5, and 6 are available at https://doi.org/10.17632/wxk9gnw22r.1. These files include details of peptide sequence, redundancy, protein assignment, raw unprocessed TMT reporter intensities and isolation specificity. The mass spectrometry proteomics data have been deposited to the ProteomeXchange Consortium (http://www.proteomexchange.org/) via the PRIDE partner repository with the dataset identifier PXD012785.

Cell Reports, Volume *27*

Supplemental Information

Quantitative Temporal Proteomic Analysis of Vaccinia Virus Infection Reveals Regulation of Histone Deacetylases by an Interferon Antagonist

Lior Soday, Yongxu Lu, Jonas D. Albarnaz, Colin T.R. Davies, Robin Antrobus, Geoffrey L. Smith, and Michael P. Weekes

Figure S1

A

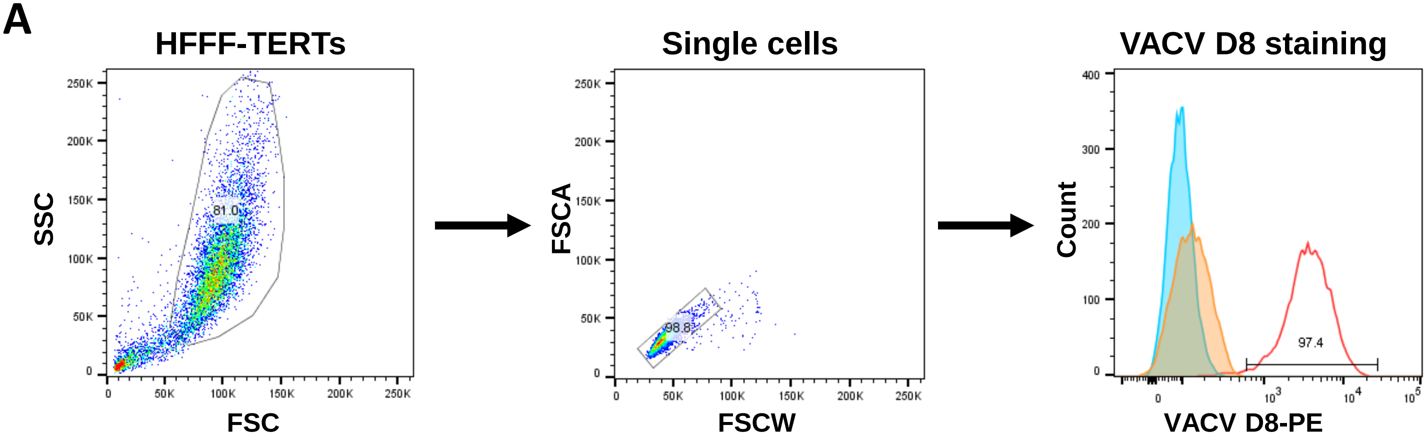

B

	WCL1	WCL2	WCL3	Any WCL	All WCL	MG / C6 data
Total peptides	120345	124779	112310			123980
Human proteins	8229	8310	8076	8991	7316	8263
Viral proteins	166	169	169	172	160	173

C

	**Sensitive Criteria** (average fold change of 2 at any time point)	**Stringent Criteria** (quantified in WCL1, 2 and 3; average fold change of 2 and a p <0.05 at any time point)
Total Proteins	8991	7316
Downregulated	265	142
Upregulated	70	4

D

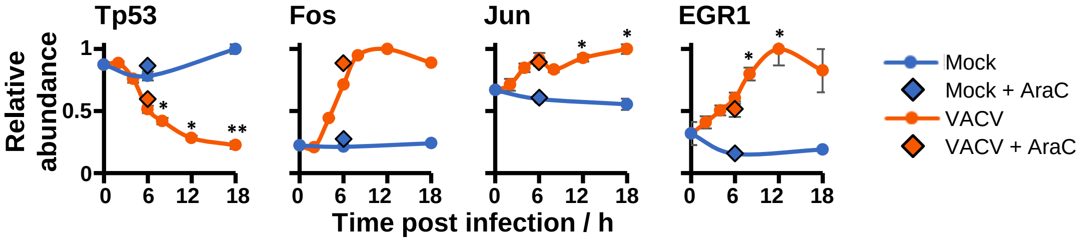

E

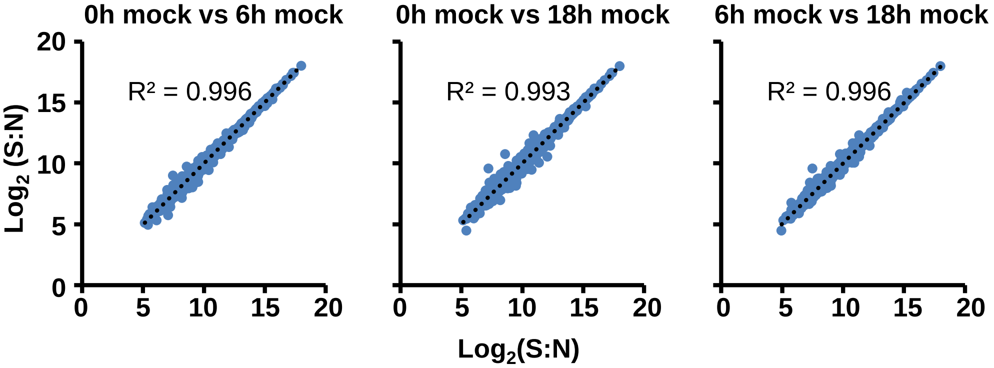

Figure S1. Technical aspects of the expreiments. related to Figures 1-5.

(A) Flow cytometry of HFFF-TERTs 12 h post infection using a mAb against late protein D8 (Parkinson and Smith, 1994) confirmed > 95% of cells were infected. A total 10,000 events were recorded, followed by sequential gating of HFFF-TERTs and single-cell populations. Isotype antibody staining of infected cells and D8 staining of non-infected cells were used to define the boundaries between "D8-positive" and "D8-negative" cells. Data shown are from experiment 1, and are representative of all three replicates.

(B) Peptides and proteins quantified in each experiment in this manuscript.

(C) Number of proteins that changed by sensitive or stringent criteria (see Figure 1C for definition of these criteria).

(D) Validation of down- or up-regulation of proteins already reported to be modulated by VACV. Data are represented as mean ± SEM; *p<0.05, *p<0.01 (see STAR Methods). Error bars and statistics are not included on the plot for Fos as this protein was not quantified in all three replicates; full data is shown in Table S1.

(E) Correspondence between mock samples. Each graph shows $\log_2$(normalised signal:noise) for every protein assessed in all three biological replicates.

Figure S2

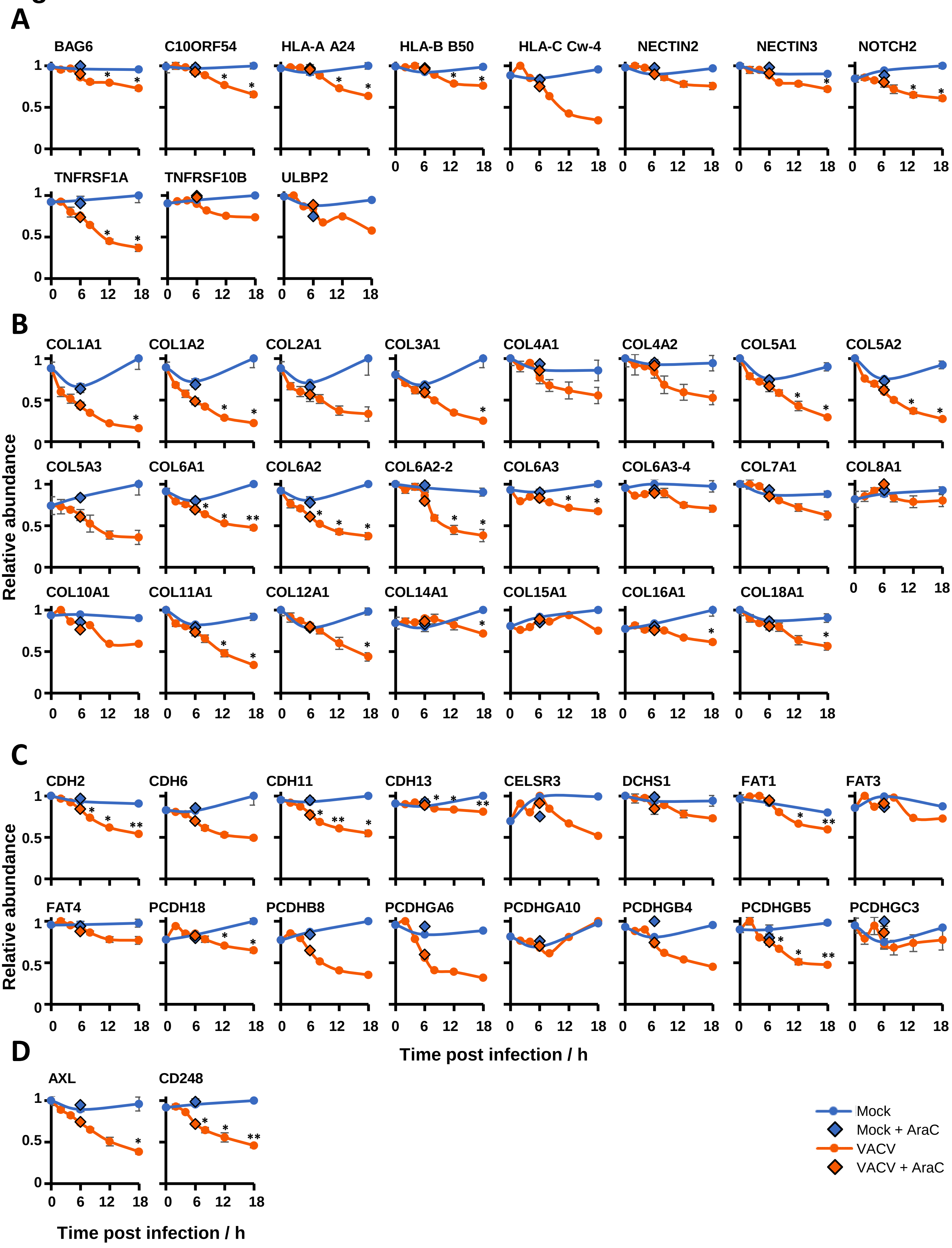
A
BAG6
C10ORF54
HLA-A A24
HLA-B B50
HLA-C Cw-4
NECTIN2
NECTIN3
NOTCH2
TNFRSF1A
TNFRSF10B
ULBP2
B
COL1A1
COL1A2
COL2A1
COL3A1
COL4A1
COL4A2
COL5A1
COL5A2
COL5A3
COL6A1
COL6A2
COL6A2-2
COL6A3
COL6A3-4
COL7A1
COL8A1
COL10A1
COL11A1
COL12A1
COL14A1
COL15A1
COL16A1
COL18A1
C
CDH2
CDH6
CDH11
CDH13
CELSR3
DCHS1
FAT1
FAT3
FAT4
PCDH18
PCDHB8
PCDHGA6
PCDHGA10
PCDHGB4
PCDHGB5
PCDHGC3
D
AXL
CD248
Relative abundance
Time post infection / h
Mock
Mock + AraC
VACV
VACV + AraC

Figure S2, Modulation of known and putative immune ligands by VACV infection, related to Figure 2. Data are represented as mean ± SEM; *p<0.05, *p<0.01 (see STAR Methods). In this figure, error bars and statistics are not included on the plots for HLA-C, TNFRSF10B, ULBP2, COL10A1, COL15A1, CELSR3, FAT3, PCDHB8, PCDHGA6, PCDHGA10, PCDHGB4 as these proteins were not quantified in all three replicates; full data is shown in Table S1.

(A) Modulation of known NK- or T-cell ligands.
(B) All collagens quantified.
(C) All protocadherins quantified.
(D) Downregulation of AXL and CD248.

Figure S3

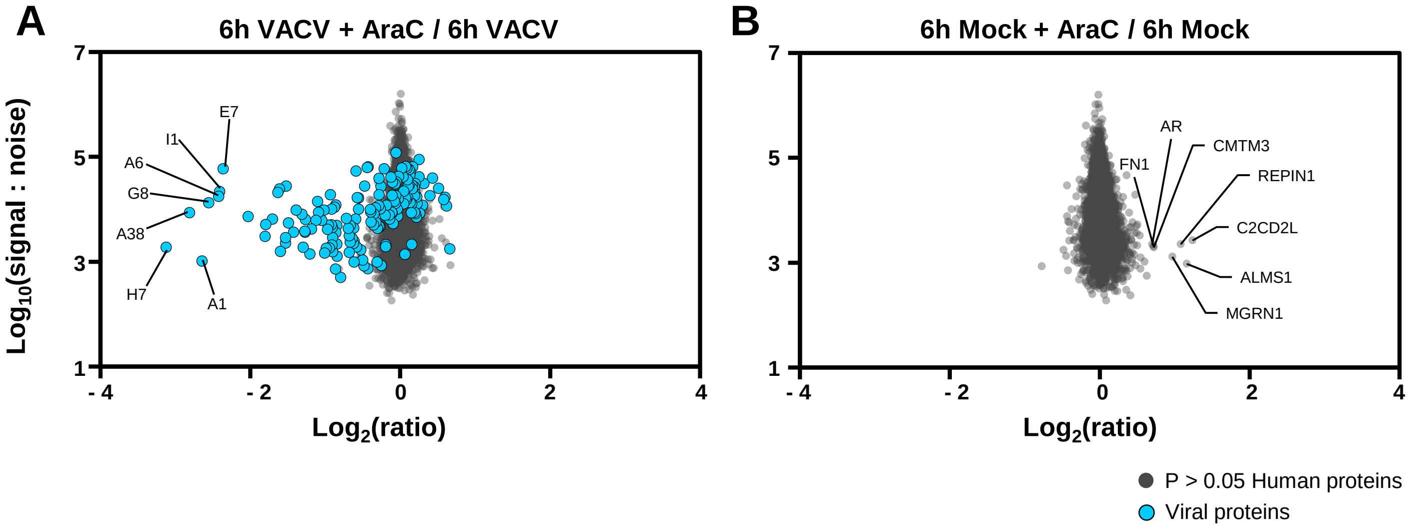

Figure S3. AraC inhibits expression of a subset of viral proteins, related to Figure 4.

(A) Scatter plot of all proteins quantified at 6 h of infection in all three replicates in the presence or absence of AraC. A Benjamini-Hochberg-corrected two-tailed t-test was used to estimate p-values.

(B) Scatter plot of all proteins quantified at 6 h of mock infection in the presence or absence of AraC. VACV proteins were quantified at the level of noise in mock samples, and are not shown.

Figure S4

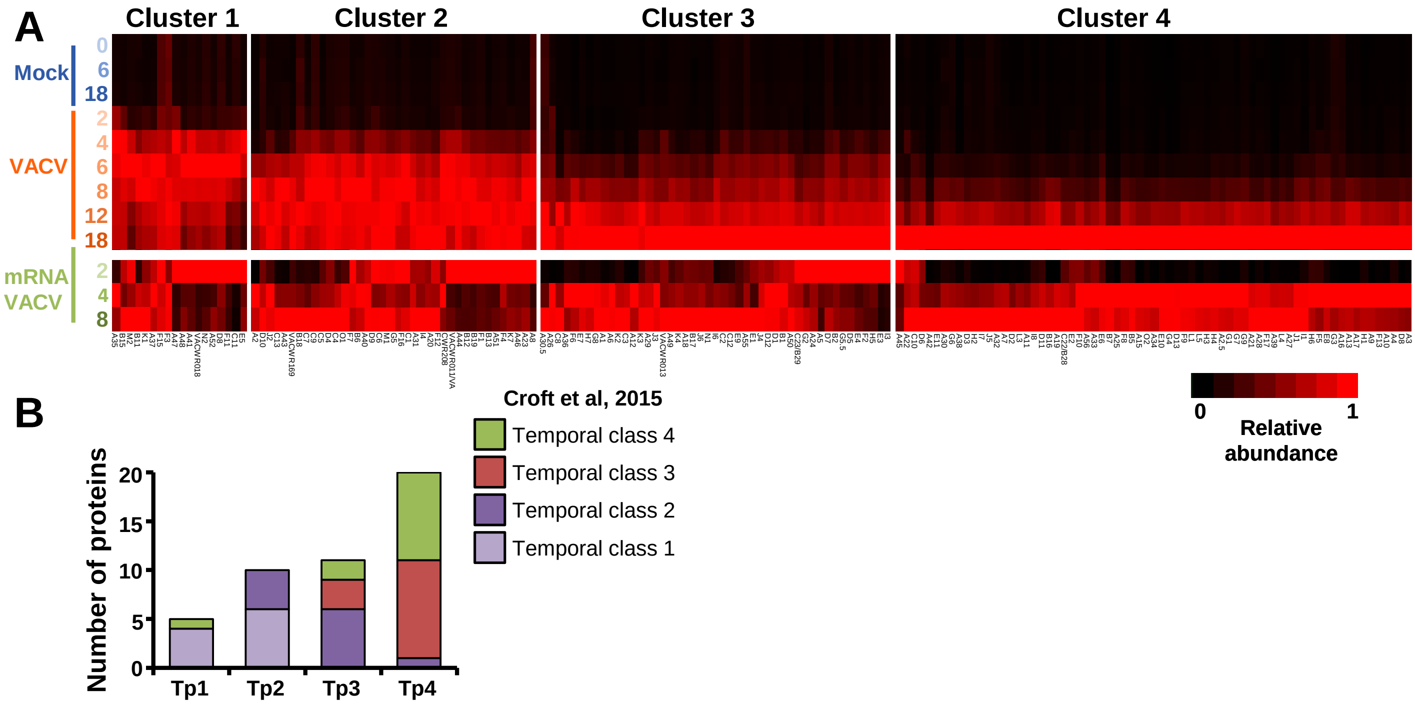

Figure S4. Comparison of viral protein data to previous studies, related to Figure 4.

(A) Comparison between temporal protein profiles (this study) and transcript expression profiles (Yang et al., 2015), grouped according to protein class. For each protein or transcript, expression was normalised to the maximum across the measured time points.

(B) Comparison of viral protein classes between this study and (Croft et al., 2015). Full data and an explanation of the consensus between the two time courses conducted by Croft et al., is included in Table S5.

Figure S5

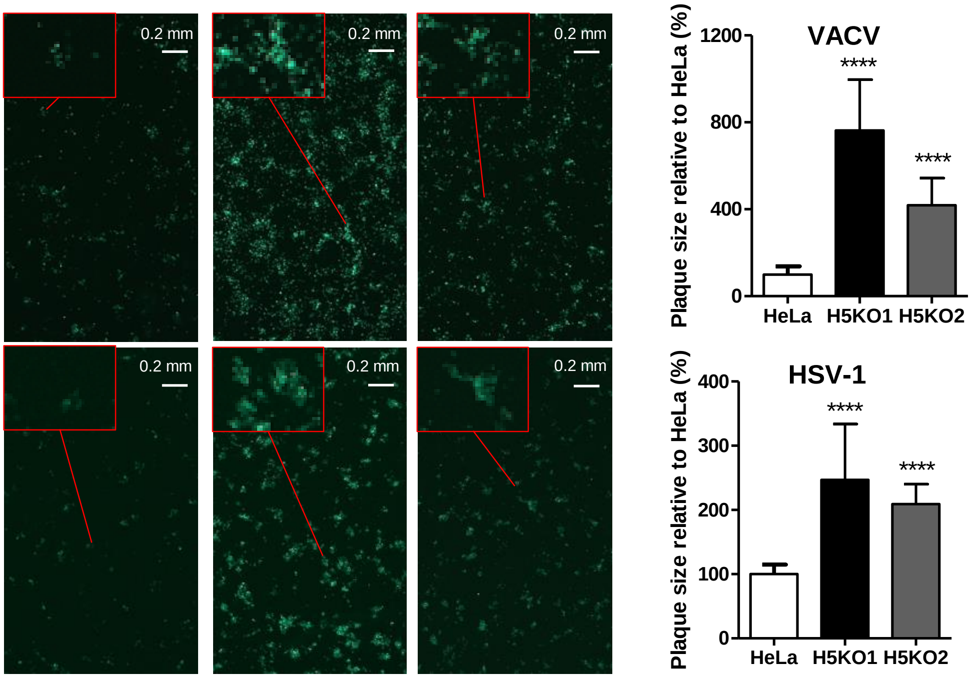

Figure S5. Enhanced spread of VACV and HSV-1 in HeLa H5KO1 and H5KO2 cells, related to Figure 7. Cells were infected with A5GFP VACV (for 2 d and VP26GFP HSV-1 for 3 d (MOI=0.001) and then imaged by fluorescence microscopy. The size of GFP infected foci (n=20) were measured for each virus and quantified as described in Materials and Methods. p-values were calculated using a two-tailed t-test. ****$p<0.0001$. The images shown are representative of 5 independent experiments.
